# Developmental patterning of peptide transcription in the central circadian clock in both sexes

**DOI:** 10.3389/fnins.2023.1177458

**Published:** 2023-05-19

**Authors:** Vania Carmona-Alcocer, Lindsey S. Brown, Aiesha Anchan, Kayla E. Rohr, Jennifer A. Evans

**Affiliations:** ^1^Department of Biomedical Science, Marquette University, Milwaukee, WI, United States; ^2^Harvard John A. Paulson School of Engineering and Applied Sciences, Harvard University, Allston, MA, United States

**Keywords:** circadian, suprachiasmatic nucleus, development, neuropeptide transcription, sex differences, spatial mapping

## Abstract

**Introduction:**

Neuropeptide signaling modulates the function of central clock neurons in the suprachiasmatic nucleus (SCN) during development and adulthood. Arginine vasopressin (AVP) and vasoactive intestinal peptide (VIP) are expressed early in SCN development, but the precise timing of transcriptional onset has been difficult to establish due to age-related changes in the rhythmic expression of each peptide.

**Methods:**

To provide insight into spatial patterning of peptide transcription during SCN development, we used a transgenic approach to define the onset of *Avp* and *Vip* transcription. *Avp-Cre* or *Vip-Cre* males were crossed to Ai9^+/+^ females, producing offspring in which the fluorescent protein tdTomato (tdT) is expressed at the onset of *Avp* or *Vip* transcription. Spatial patterning of *Avp-tdT* and *Vip-tdT* expression was examined at critical developmental time points spanning mid-embryonic age to adulthood in both sexes.

**Results:**

We find that *Avp-tdT* and *Vip-tdT* expression is initiated at different developmental time points in spatial subclusters of SCN neurons, with developmental patterning that differs by sex.

**Conclusions:**

These data suggest that SCN neurons can be distinguished into further subtypes based on the developmental patterning of neuropeptide expression, which may contribute to regional and/or sex differences in cellular function in adulthood.

## Introduction

Daily rhythms in mammals are programmed by the circadian timekeeping system (Mohawk et al., 2012), which ensures that behavior and physiology are well matched to environmental conditions over the solar day. In nearly every biological system, cell physiology is modulated by autoregulatory genetic feedback loops controlling circadian rhythms in gene expression (Buhr and Takahashi, 2013). At the system level, clock tissues in the body are coordinated by a central clock in the suprachiasmatic nucleus (SCN), which is necessary for daily rhythms in behavior and physiology (Hastings et al., 2018). As the central pacemaker, the SCN processes photic inputs from the retina, sustains tissue-level rhythms through local communication, and provides outputs to coordinate cellular rhythms in downstream targets. Neural network mechanisms that support SCN timekeeping are essential for achieving internal and external coordination of the circadian system in an ever-changing environment.

The SCN is a heterogenous network of cellular clocks that displays self-sustained circadian rhythms in metabolism, electrical activity, gene/protein expression, and peptide release (Hastings et al., 2018). SCN neurons express the neurotransmitter GABA and can be distinguished into different subpopulations based on peptide expression (Antle et al., 2003). Two types of SCN neurons have been studied in mammals in depth (Abrahamson and Moore, 2001; Moore et al., 2002; Ono et al., 2021). Located in the SCN shell and core respectively, AVP and VIP neurons provide network signals that regulate daily rhythms in behavior and physiology (Vosko et al., 2007; Kalsbeek et al., 2010; Ono et al., 2021). In addition to regional patterns of peptide expression, SCN neurons display cellular rhythms with spatial gradients that repeat across the network each circadian cycle (Hamada et al., 2004; Evans et al., 2011; Enoki et al., 2012; Brancaccio et al., 2013). Spatial gradients in clock function are stereotyped across individual animals, are evident in a variety of cellular processes, and can be modulated by experience (Inagaki et al., 2007; Evans et al., 2013). How neural identity maps onto differences in cellular function in the SCN network is a key question in the field.

The importance of the SCN clock during adulthood is well established, but the process by which SCN circuits form is not fully understood (Landgraf et al., 2014; Bedont and Blackshaw, 2015; Carmona-Alcocer et al., 2020). Across mammalian species, SCN neurogenesis occurs over the third to fourth quarter of gestation (Shimada and Nakamura, 1973; Altman and Bayer, 1978; Davis et al., 1990; Antle et al., 2005; Kabrita and Davis, 2008). The onset of daily rhythms in SCN activity has been detected as early as the end of neurogenesis and as late as the first few days after birth (Reppert, 1992; Shimomura et al., 2001; Sladek et al., 2004; Ohta et al., 2006; Wreschnig et al., 2014; Carmona-Alcocer et al., 2018). Despite these early milestones, postnatal development is critical for SCN circuit formation (Landgraf et al., 2014; Bedont and Blackshaw, 2015; Carmona-Alcocer et al., 2020). Both *Avp* and *Vip* transcripts are detected in the mouse SCN during late embryonic development (Okamura et al., 1983; Vandunk et al., 2011), but transcript and peptide levels increase over the first 3 weeks after birth (Hyodo et al., 1992; Ban et al., 1997; Herzog et al., 2000). Previous work suggests that the roles of AVP and VIP in the regulation of SCN function vary over development (Wreschnig et al., 2014; Ono et al., 2016; Carmona-Alcocer et al., 2018; Mazuski et al., 2020), but how these peptide circuits mature remains unclear.

One outstanding question concerns spatial patterning of SCN circuits during development. Spatiotemporal gradients in SCN neurogenesis have been reported, with SCN core neurons appearing before those in the SCN shell in mice, rats, and hamsters (Altman and Bayer, 1978; Davis et al., 1990; Antle et al., 2005; Kabrita and Davis, 2008). In the mouse, SCN shell neurons are generated in the middle-posterior regions before those in the anterior pole (Okamura et al., 1983; Kabrita and Davis, 2008). These studies suggest that SCN neurons in different regions of the network develop at different times, but it remains unclear if spatial patterning occurs for other milestones in cellular development (e.g., differentiation). Interestingly, previous work suggests that the onset of *Vip* transcription occurs in two distinct subclusters of SCN neurons that differ in spatial location and cellular function in adulthood (Ban et al., 1997). One obstacle in understanding SCN peptide development is that rhythms in SCN transcripts can change as the network matures (Isobe and Muramatsu, 1995; Ban et al., 1997; Shimomura et al., 2001; Houdek and Sumova, 2014). The resulting need to conduct a circadian time course at each developmental age has limited insight into spatial patterning during SCN development.

Here we use a genetic approach to test if SCN neurons display spatial patterning of peptide transcription during development. This approach uses Cre to permanently label cells with a fluorescent reporter at the time of *Avp* and *Vip* transcription (Harris et al., 2014; Taniguchi, 2014), thus circumventing the need to conduct a circadian time course to detect expression of the peptide itself. Using this genetic approach, we tracked *Avp* and *Vip* transcription across the entire SCN at key stages of pre-and post-natal development. We find that genetically labeled cells in each peptide class appear in spatially distinct subclusters over development. In addition, we find that biological sex influences developmental patterning of *Avp* and *Vip* labeling in a manner that differs for each SCN peptide class. Collectively, these data suggest that SCN neurons can be distinguished into further subclasses based on developmental patterning of neuropeptide transcription.

## Materials and methods

### Mice lines and general husbandry

Mice were bred and raised under a 24-h light–dark cycle with 12 h of light and 12 h of darkness [LD12:12: lights off: 1800 CST defined as Zeitgeber Time 12 (ZT12)]. Throughout life, ambient temperature was maintained at 22°C ± 2°C, and mice had *ad libitum* access to water and food (Teklad Rodent Diet 8,604). These studies used mice derived from crossing *Ai9*^+/+^ females (Madisen et al., 2010) with *Avp*-IRES2-Cre^+/−^ males (Harris et al., 2014), JAX# 023530, C57Bl/6 background) or *Vip*-IRES-Cre^+/+^ males (Taniguchi, 2014), JAX# 010908, C57Bl/Jx129S background). In the heterozygous progeny of this cross (i.e., *Avp-Cre^+/−^*; *Ai9^+/−^* and *Vip-Cre^+/−^*; *Ai9^+/−^*), Cre recombinase is expressed under the *Avp/Vip* promoter, causing cell-specific expression of the red fluorescent protein, tdTomato (tdT) at the onset of peptide transcription. For convenience, we refer to these as *Avp-tdT* and *Vip-tdT* mice. All procedures were conducted according to the NIH Guide for the Care and Use of Animals and were approved by the Institutional Animal Care and Use Committees at Marquette University.

### Experimental breeding

To genetically label *Avp and Vip* neurons over specific developmental ages, male *Avp-Cre* or *Vip-Cre* mice were paired overnight with nulliparious female *Ai9^+/+^* mice. On the morning following cohabitation, successful mating was verified by the presence of vaginal plugs and designated Embryonic Day 1 (E01). Pregnant dams were tracked throughout pregnancy, and the day of birth was designated Postnatal Day 0 (P00). Sex and genotype of offspring were determined by PCR amplification of *Sly/Xlr* (McFarlane et al., 2013) and *Cre^+/−^* (Jackson Laboratory, oligo primers # 18475, 18,474, 10,362), respectively. Both male and female mice were used in all experiments, with biological sex confirmed by genotyping (McFarlane et al., 2013).

### Brain collection, tissue processing, and microscopy

To evaluate specificity of labeling, brains were collected from *Ai9*^+/+^, *Avp-tdT* and *Vip-tdT* mice of both sexes and sectioned in the coronal plane (40 μm) prior to mounting onto microscope slides for cell counting. To evaluate the correspondence between tdT labeling and peptide expression in adulthood, *Avp-tdT* and *Vip-tdT* mice of both sexes (*n* = 4-5/sex/genotype, P84, 22 weeks of age) received 1 μl colchicine injection into the third ventricle (0.5 μL/min) to slow microtubule transport and measure cumulative peptide expression over the circadian cycle. Brains were collected 48 h later (ZT06) and fixed in 4% paraformaldehyde overnight, cryoprotected in 20% sucrose for 4 days, and then sectioned in the coronal plane (25 μm). Free-floating slices were washed 6 times in PBS, blocked for 1 h in normal donkey serum, incubated for 48 h at 4°C with primary antibodies (Rabbit anti-AVP, Millipore AB1565, 1:1 K; Rabbit anti-VIP, Sigma HPA017324, 1:500), washed 6 times in PBS, incubated for 2 h at room temperature with secondary antibodies (Alexa Fluor 488, Donkey anti-rabbit, JIR 711–545-152, 1:500), and then washed 6 times in PBS before mounting in Prolong Anti-Fade medium with DAPI (Thermo Fisher, Cat# P36935) and cover slipped. For each experiment, slices were imaged by collecting 10X Z-stack images on a Nikon A1R+ confocal microscope (Nikon Instruments, Melville, NY, United States). The anterior, middle, and posterior SCN slice was identified for each sample and used for data analyses. Using ImageJ, a hyperstack projection of the Z-stack for each slice from each sample was created, and the total number of tdT+ and/or AVP/VIP+ cells was counted using the 3D Object Counter module.

To evaluate developmental patterns of *Avp-tdT* and *Vip-tdT* expression, brains were collected at E16, E18, E19, P01, P03, P05, P10 or P84 (i.e., Adult, *n* = 3–7 mice/sex/genotype, at least 2 litters collected at each age). For embryonic ages, pregnant females were anesthetized with isoflurane and euthanized by cervical dislocation before pups were extracted from the uterus and decapitated. Postnatal mice were euthanized by decapitation, whereas adult mice were anesthetized and euthanized as described for dams. Brains were collected in the middle of the photophase (ZT06), except E19 brains were collected 1 h before lights-off. Brains were fixed in 4% paraformaldehyde overnight at 4°C, cryoprotected in 20% sucrose for 48 h and 30% sucrose for 72 h at 4°C, then sectioned in the coronal plane (40 μm). All slices through the entire SCN were retained as one series and thaw-mounted onto microscope slides or saved as free-floating slices (P84). Nuclear staining was achieved by embedding slices in DAPI-containing mounting media (Abcam, Cat# ab104135) before cover-slipping. As described above, confocal images of tdT expression were collected. Using ImageJ, SCN images were aligned across samples in the XY plane using the Python OpenCV package and verified manually using SCN DAPI-determined boundaries. tdT+ cells were counted using a hyperstack as above, and the XYZ location of each cell was recorded (Supplementary Figure S1). Each SCN slice was mapped to the corresponding slice in the adult data based on preserved morphology across ages (Supplementary Figures S2A,B). Sex did not influence SCN area over development (Supplementary Figure S2C). tdT+ cells were counted using a hyperstack as above, and the XYZ location of each cell was recorded (Supplementary Figure S1). Cell counts were analyzed based on anteroposterior SCN region (anterior, middle, posterior SCN). In addition, cell clusters were identified using k-means clustering (Python scikit-learn), with the optimal number of clusters determined by the location of the elbow in the sum of squared distances (Nugent and Meila, 2010). At each developmental timepoint, cells were assigned to one of the spatial clusters identified in P84 adult samples. To visualize cellular density in different SCN regions, cellular coordinates were used to determine the number of neighboring cells within a 50 μm radius for each sample (Supplementary Figures S3A–C).

### Data analyses

Statistical analyses were performed with JMP software (SAS Institute). Data are represented in figures and tables as mean ± SEM. When datasets contained within-subject factors (Slice Position, Cell Cluster), a mixed linear model was used to parse out random effects driven by individual differences among mice. When models only contained between-subject factors (Sex, Cell Type, Age), a full-factorial ANOVA was used to assess main effects and interactions. *Post-hoc* tests were performed with Tukey’s HSD or Least Square Mean contrasts to control for family-wise error. Statistical significance was set at *p* < 0.05.

## Results

### Genetic approach for labeling SCN neurons by neuropeptide class

**Figure 1:**
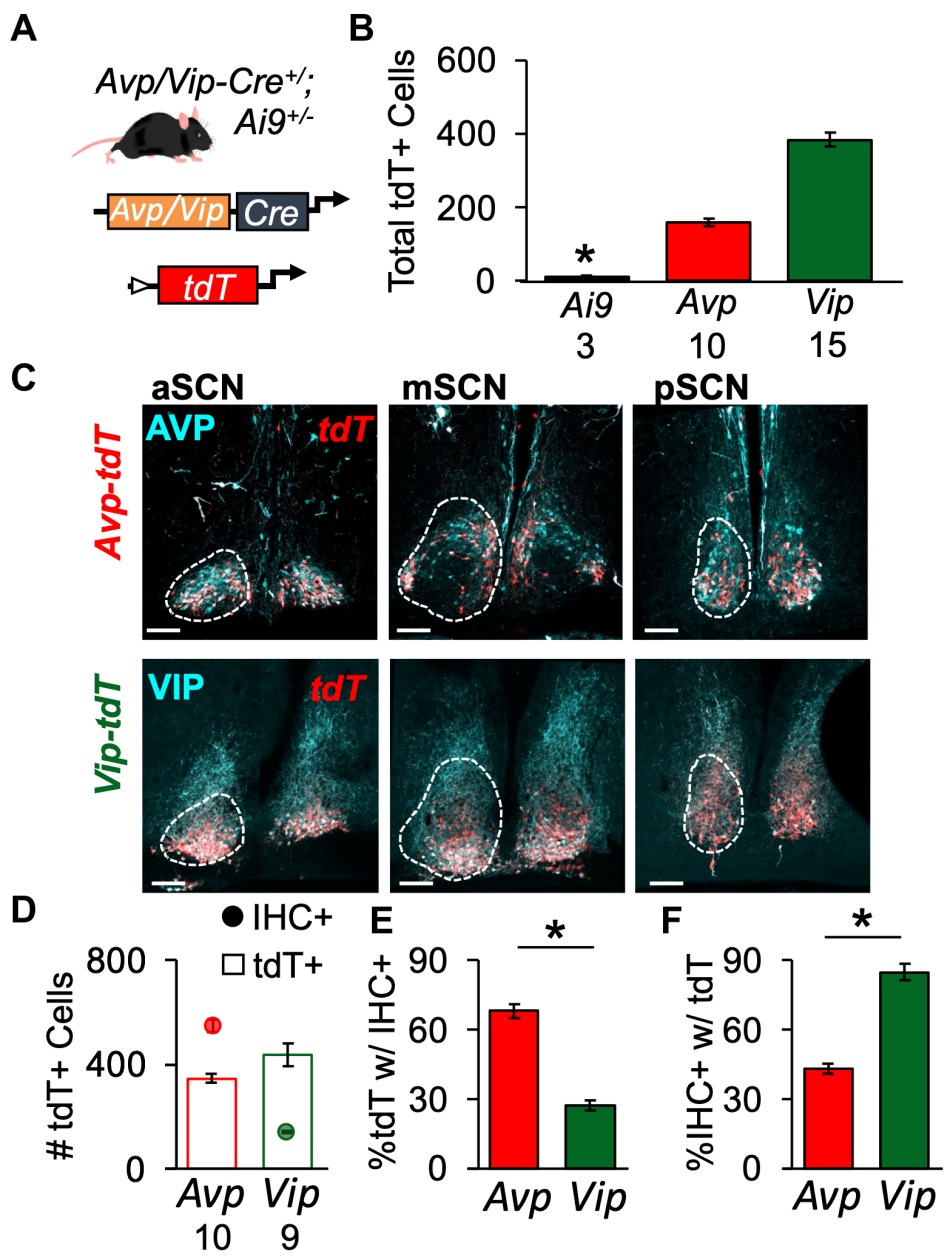
Genetic strategy to label spatial location of SCN peptide classes. **(A)** Schematic illustrating genetic approach to label *Avp-* and *Vip-*expressing SCN neurons with tdT. **(B)** tdT labeling is Cre-dependent in the SCN. Also see Supplementary Figure S4. Cre: *F*(2,22) = 24.1, *p* < 0.0001; Sex: *F*(1,22) = 0.3, *p* > 0.5; Cre*Sex: *F*(2,22) = 0.6, *p* > 0.5. **(C)** Representative SCN slices illustrating tdT and AVP/VIP expression in a male *Avp-tdT* or *Vip-tdT* SCN. Scale bars = 100 μm. **(D)** Total number of labeled neurons collapsed across three SCN slices. AVP/VIP IHC: Cre: *F*(1,15) = 175, *p* < 0.0001; Sex: *F*(1,15) = 0.2, *p* > 0.6; Cre*Sex: *F*(1,15) = 0.3, *p* > 0.5. tdT+: Cre: *F*(1,15) = 4.12, *p* = 0.06; Sex: *F*(1,15) = 2.3, *p* > 0.1; Cre*Sex: *F*(1,15) = 2.9, *p* > 0.1. **(E)** More *Avp-tdT+* neurons express AVP compared to *Vip-tdT+* neurons that express VIP. Cre: *F*(1,15) = 112.5, *p* < 0.0001; Sex: *F*(1,15) = 3.2, *p = 0.09*; Cre*Sex: *F*(1,15) = 0.1, *p* > 0.8. **(F)** More *Vip-tdT+* express tdT compared to *Avp-tdT+* neurons. Cre: *F*(1,15) = 105.8, *p* < 0.0001; Sex: *F*(1,15) = 1.6, *p* > 0.2; Cre*Sex: *F*(1,15) = 0.2, *p* > 0.6. Numbers below *x*-axis in panels **(B,D)** indicate sample sizes for each group. Contrasts comparing genotype, *p < 0.05.

To evaluate spatial patterning of SCN development, we employed a genetic strategy (Figure 1A). Driven by the *Avp-* or *Vip-*promoter, Cre recombinase induced tdTomato (tdT) expression in *Avp-tdT* and *Vip-tdT* mice. As expected, tdT expression in the SCN was Cre-dependent, with very little recombination in *Ai9^+/+^* mice (Figure 1B, Supplementary Figure S4). Next, co-expression of tdT and AVP/VIP peptide expression was evaluated in adult mice using *in vivo* intracranial colchicine injections and immunohistochemistry (Figures 1C–F, Supplementary Figures S5A–D). AVP-IHC+ neurons outnumbered VIP-IHC+ neurons (Figure 1D, Cre: *F*(1,15) = 175, *p* < 0.0001), as expected based on previous work in the mouse (Abrahamson and Moore, 2001). However, the number of *Avp-tdT+* and *Vip-tdT+* SCN cells were more similar [Figure 1D, Cre: *F*(1,15) = 4.1, *p* = 0.06]. Approximately 30% *Vip-tdT+* neurons were co-labeled by IHC, compared to 70% of *Avp-tdT+* neurons [Figure 1E, Cre: *F*(1,15) = 112.5, *p* < 0.0001]. On the other hand, over 80% of VIP-IHC+ neurons were co-labeled with tdT, compared to only 43% of AVP-IHC+ neurons [Figure 1F, Cre: *F*(1,15) = 105.8, *p* < 0.0001]. Failure of Cre-mediated recombination in the *Avp-tdT* model appeared to be highest in the dorsal middle SCN (Figure 1C, Supplementary Figures S5A,E). Importantly, sex did not influence measures of tdT/AVP/VIP labeling or co-expression (Supplementary Figures S5C–F). These results indicate that this genetic approach does not fully capture peptide expression in the adult SCN, but that tdT can be used in both sexes.

### Mapping *Avp-tdT*+ and *Vip-tdT*+ neurons in the adult SCN

**Figure 2:**
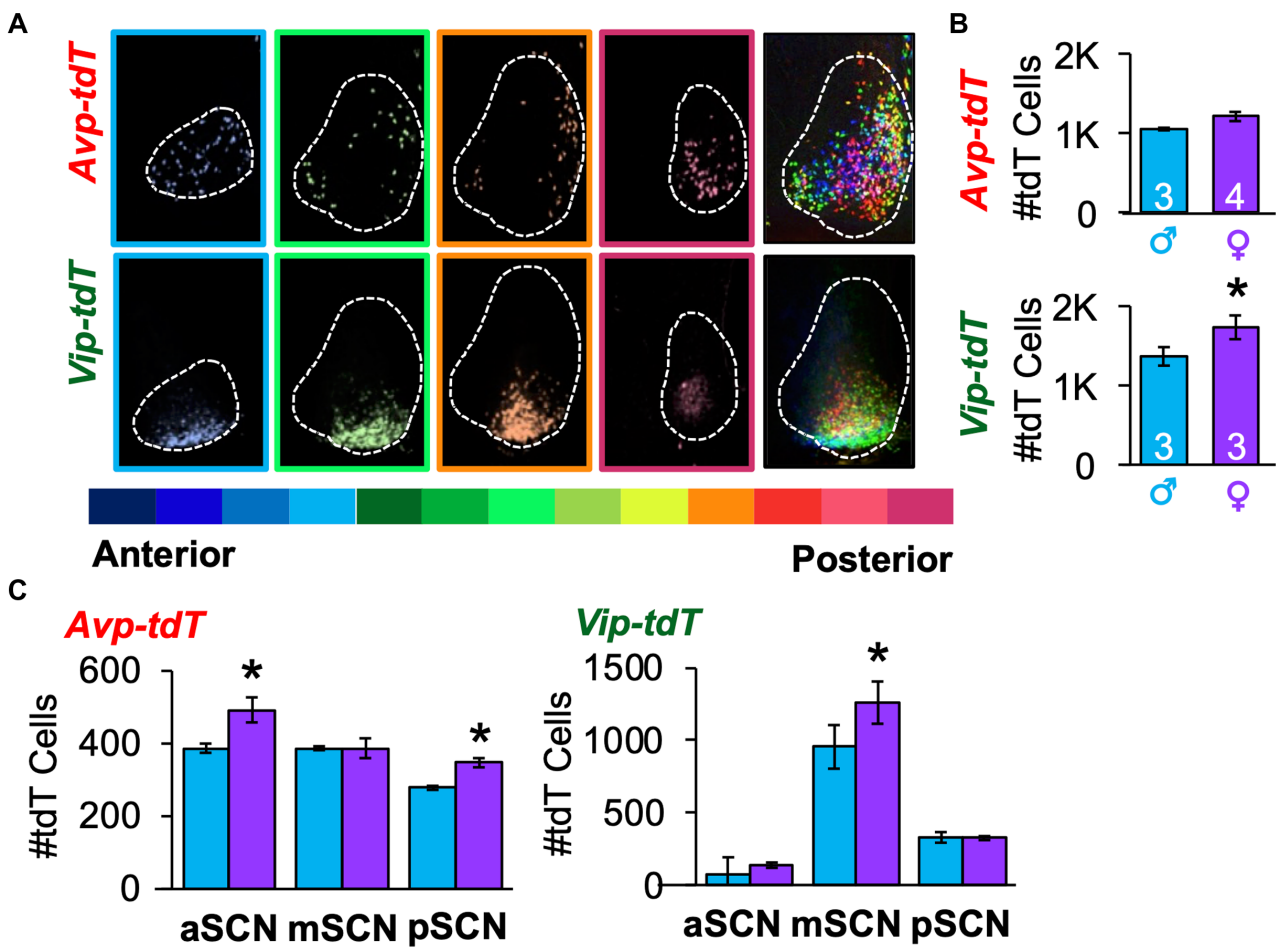
Spatial mapping of SCN neurons in each peptide class in adulthood. **(A)** A representative subset of SCN slices collected through the anteroposterior axis from a female *Avp-tdT* or *Vip-tdT* mouse. Each slice is color-coded by slice position, with cells in all SCN slices superimposed in rightmost panel. The full set of SCN slices from this mouse is illustrated in Supplementary Figure S1A. **(B)** Total number of SCN *Avp-tdT+* and *Vip-tdT+* neurons in each sex. Cre: *F*(1,9) = 18.1, *p* < 0.005, Sex: *F*(1,9) = 7.3, *p* < 0.05; Sex*Cre: *F*(1,9) = 1.1, *p* > 0.3. **(C)** Sex influences the number of *Avp-tdT+* and *Vip-tdT+* neurons in different anteroposterior SCN regions. *Avp-tdT* - Sex: *F*(1,5) = 5.6, *p* = 0.06; Position: *F*(2,10) = 25.1, *p* = 0.0001; Sex*Position: *F*(2,10) = 4.5, *p* < 0.05. *Vip-tdT* – Sex: *F*(1,4) = 3.6, *p* > 0.1; Position: *F*(2,8) = 63.3, *p* < 0.0001; Sex*Position: *F*(2,8) = 1.5, *p* > 0.2. aSCN, mSCN, and pSCN: Anterior, middle, and posterior SCN. Contrasts comparing male and female data for each cell type, *p < 0.05.

As a next step toward constructing a developmental atlas, we mapped the spatial location of *Avp-tdT* and *Vip-tdT* SCN neurons in adulthood using a more comprehensive approach. All *Avp-tdT+* and *Vip-tdT+* cells were counted throughout the anteroposterior SCN in each sex (Figure 2A, Supplementary Figure S1). When counted across all SCN slices, *Vip-tdT+* cells outnumbered *Avp-tdT+* cells [Figure 2B, Cre: *F*(1,9) = 18.1, *p* < 0.005], with more *Vip-tdT*+ cells in females than males [Figure 2B, Sex: *F*(1,9) = 7.3, *p* < 0.05, Contrasts, *p* < 0.05]. When parsed by SCN slice position, females displayed more *Avp-tdT+* cells than males in the anterior and posterior SCN, and females displayed more *Vip-tdT+* cells than males in the middle SCN (Figure 2C, Contrasts, *p < 0.05*).

**Figure 3:**
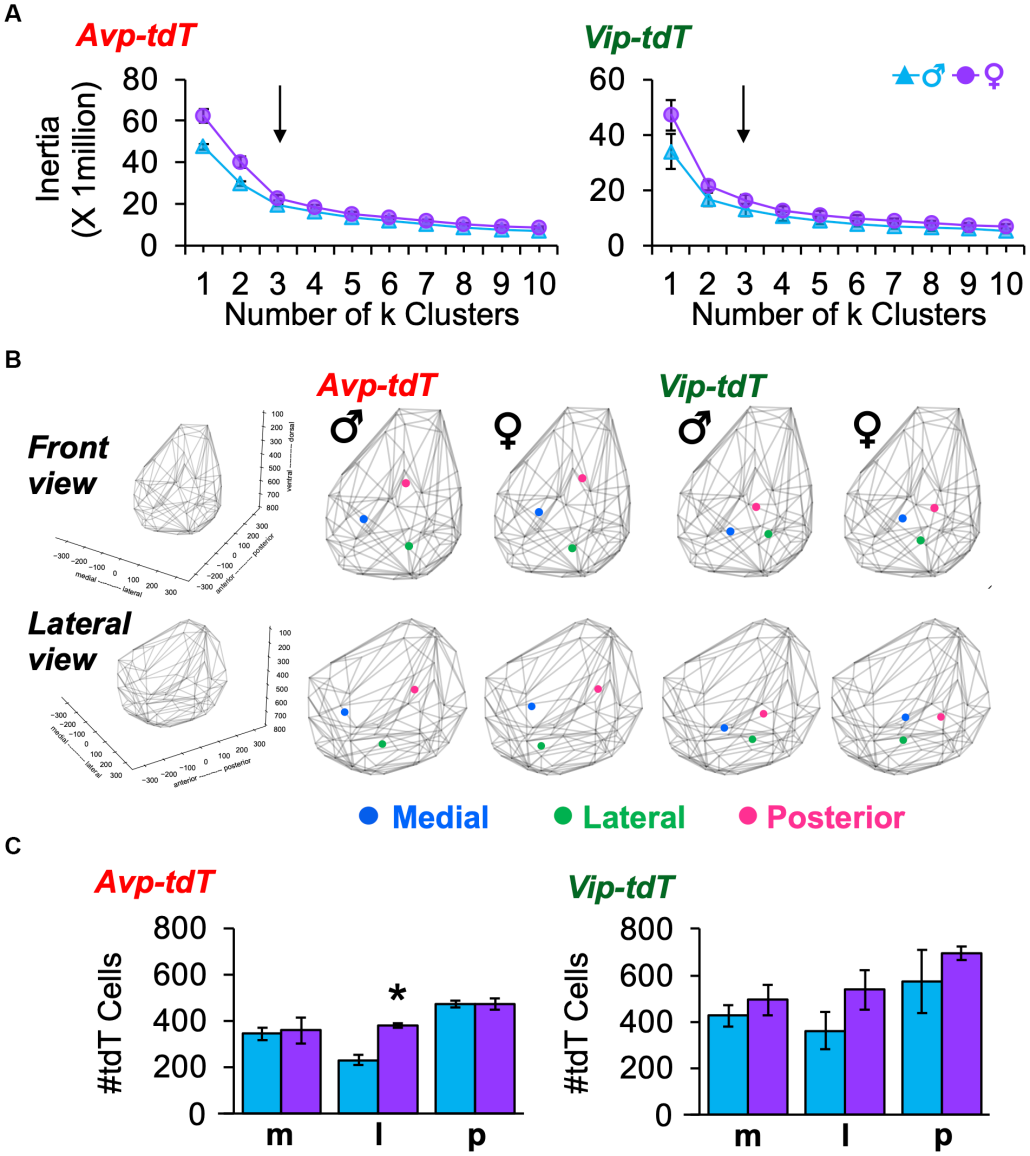
K means clustering of SCN neurons in each peptide class in adulthood. **(A)** Elbow plots illustrating measures of cell dispersion in each sex. Arrow indicates optimal number of clusters. **(B)** Spatial location of cluster centers in each sex in the front and lateral views. Lattice frames illustrate SCN boundaries determined using the position of all tdT identified cells observed across adult samples of both genotypes. **(C)** Number of labeled cells in each cluster divided by sex. Cre: *F*(1, 9) = 18.1, *p* < 0.005, Cluster: *F*(2,18) = 10.4, *p* = 0.001, Sex: *F*(1,9) = 7.3, *p* < 0.05, Cell*Cluster: *F*(2,18) = 0.2, *p* > 0.8, Cell*Sex: *F*(1,9) = 1.1, *p* > 0.3, Cluster*Sex: *F*(2,18) = 1.3, *p* > 0.3, Cell*Cluster*Sex: *F*(2,18) = 0.2, *p* > 0.8. m, medial; l, lateral; p, posterior. Contrasts comparing male and female data for each cell type, *p < 0.05.

To complement anatomical division of anteroposterior regions, we used k means clustering based on the cellular coordinates for each sample. For both cell types and sexes, the best fit was achieved by *k* = 3 spatial clusters, as determined by the elbow location for total cell dispersion (i.e., Inertia, Figure 3A) and cell dispersion normalized to the total cell counts/sample (Distance, Supplementary Figure S6A). Spatial mapping of k means revealed one posterior cluster and two clusters that were positioned more anterior, which differed in lateral-medial location (Figure 3B). As expected, there was greater dispersion of *Avp-tdT+* than *Vip-tdT+* neurons at *k* = 3 (Figure 3B, Supplementary Figure S6B), with differences in both inertia and distance [Inertia-Cre: *F*(1,9) = 17.5, *p* < 0.005; Distance-Cre: *F*(1,9) = 17.5, *p* < 0.005, Contrasts, *p* < 0.05]. There were no significant sex differences in cell dispersion [Inertia-Sex: *F*(1,9) = 4.8, *p = 0.06*; Distance- Sex: *F*(1,9) = 0.1, *p* > 0.7]. Nevertheless, more subtle sex differences were detected in the number and location of cells in specific clusters (Figures 3B,C). Specifically, females displayed a larger number of lateral *Avp-tdT+* cells relative to males (Figure 3C, Contrasts, *p* < 0.01), and the lateral cluster for both cell types was positioned more anterior in the female SCN (Figure 3B).

**Figure 4:**
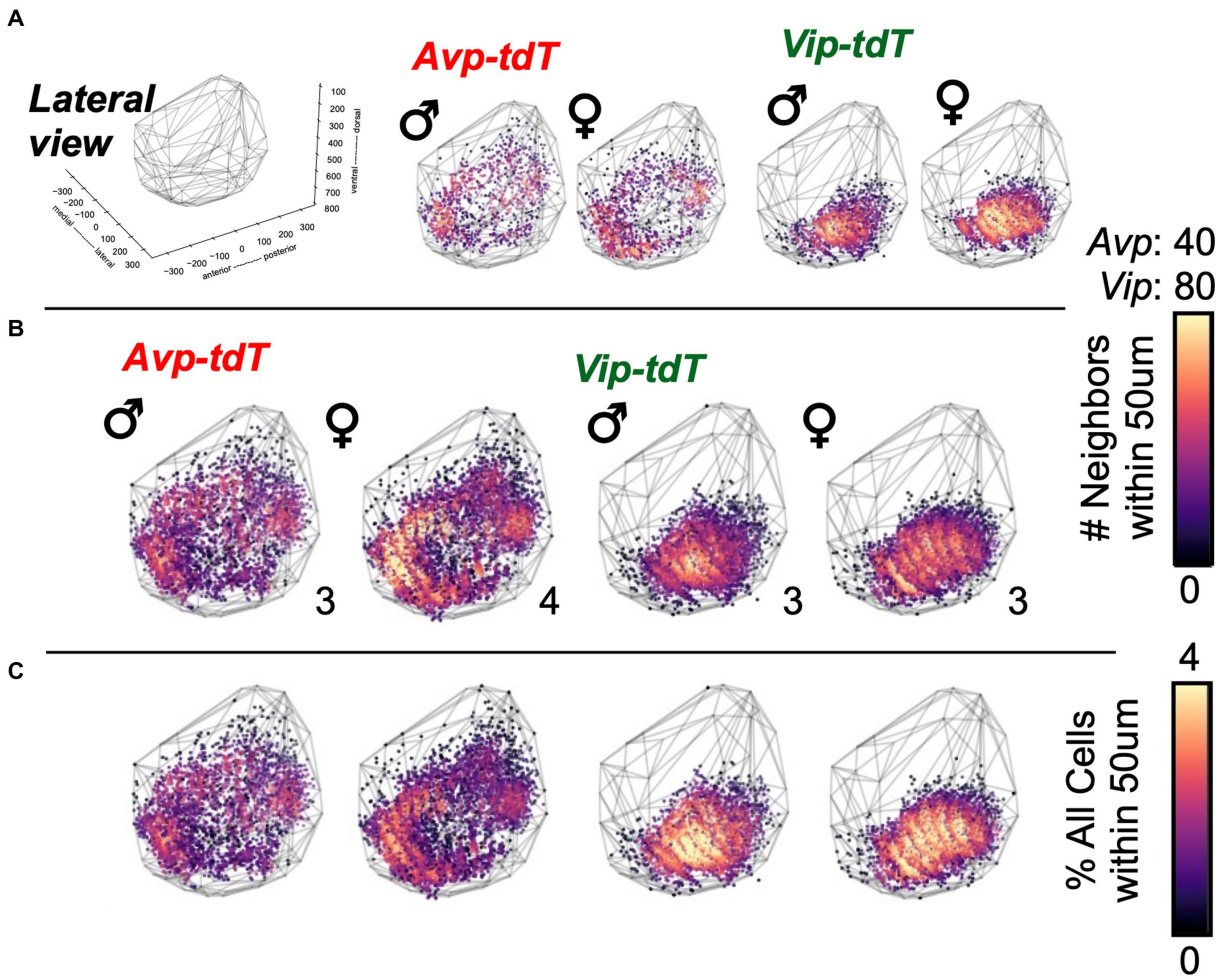
Cell density plots for SCN neurons in each peptide class in adult males and females. **(A)** Representative samples illustrating cell density in individual mice of each sex. All samples are illustrated in Supplementary Figures S3B,C. **(B–C)** Cell density plots aggregated across samples illustrating total number of neighboring cells **(B)** and the percentage of neighboring cells normalized to total SCN cells in each sample **(C)**. Number in bottom right corner for each map indicates the number of aggregated samples. Other orientations are illustrated in Supplementary Figures S7–S8 and Videos S1–S4.

To evaluate spatial patterns of cell density, next we mapped the number of neighboring cells within a 50 μm radius of each cellular coordinate (Figure 4A, Supplementary Videos S1–S4). Cell density maps were aggregated for all samples, with and without normalization to the total number of cells in each sample (Figures 4B,C, Supplementary Figures S7, S8). For both cell types, the overall morphology was similar across sex (Figures 4B,C, Supplementary Figures S7, S8). Compared to males, between-sample variability in cell density and total cell counts was larger in female *Avp-tdT* SCN neurons [Supplementary Figure S3B, Levene’s test *F*(1,5) = 10.52, *p* < 0.05], and *Avp-tdT*+ cell density was similar when normalized to the total number of cells in each sample (Figures 4B,C, Supplementary Figures S7, S8). For *Vip-tdT+* neurons, variability in cell density and total cell counts did not differ by sex [Supplementary Figure S3C, Levene’s test *F*(1,4) = 0.38, *p* > 0.7]. Collectively, these results suggest that spatial patterning of cellular density for *Avp-tdT+* and *Vip-tdT+* populations does not markedly differ between male and female SCN in adulthood.

### SCN development of *Avp-tdT* and *Vip-tdT* expression

**Figure 5:**
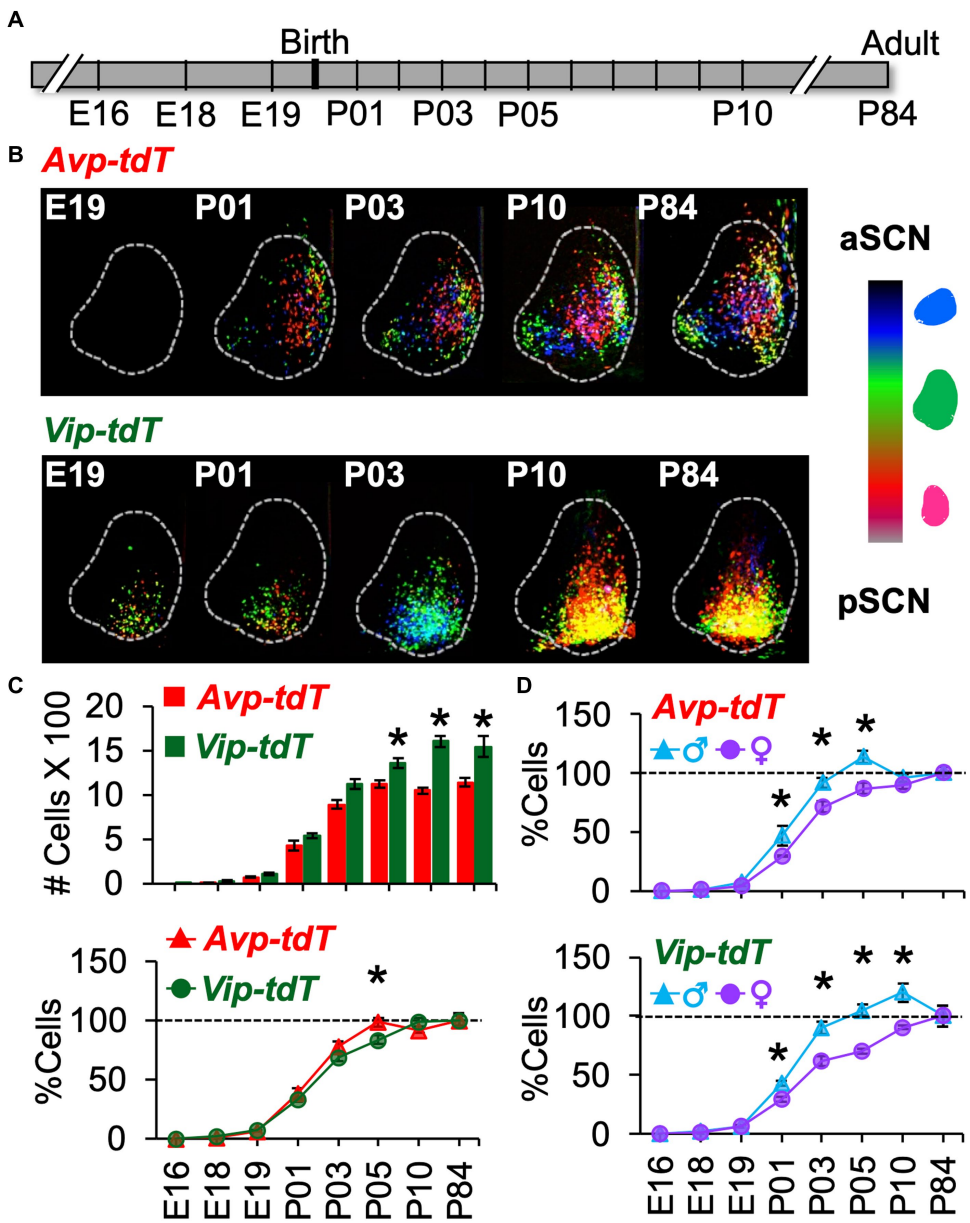
Developmental appearance of SCN *Avp-tdT*+ and *Vip-tdT*+ neurons. **(A)** Timeline illustrating ages of tissue collection. Breeding and pup development data are in Supplementary Figure S9. **(B)** Labeled cells aggregated across SCN slice positions for a representative mouse from each age group illustrating progressive appearance of *Avp-tdT+* and *Vip-tdT+* cells during postnatal development. **(C)** Top: Total number of SCN *Avp-tdT+* and *Vip-tdT+* cells across development. Cre: *F*(1,99) = 89.6, *p* < 0.001, Age: *F*(7,99) = 411.9, *p* < 0.0001, Sex: *F*(1,99) = 0.7, *p* = 0.4, Cre*Age: *F*(7,99) = 11.3, *p* < 0.0001, Cre*Sex: *F*(1,99) = 0.01, *p* > 0.9, Age*Sex: *F*(7,99) = 5.2, *p* < 0.0001, Cre*Age*Sex: *F*(7,99) = 0.9, *p* > 0.5. Bottom: To compare developmental patterns across cell type, cell counts at each age were expressed as a percent relative to the number of labeled cells in adults. Cre: *F*(1,99) = 0.2, *p* > 0.6, Age: *F*(7,99) = 409.4, *p* < 0.0001, Sex: *F*(1,99) = 50.2, *p* < 0.0001, Cre*Age: *F*(7,99) = 2.9, *p* < 0.01, Cre*Sex: *F*(1,99) = 1.6, *p* > 0.2, Age*Sex: *F*(7,99) = 8.3, *p* < 0.0001, Cre*Age*Sex: *F*(7,99) = 1.1, *p* > 0.3. Total cell counts divided by sex, with magnification of E18-P01 data, are in Supplementary Figure S10A. **(D)** Percent labeled cells at each age divided by sex and cell type. *Avp-tdT+*: Age: *F*(7,47) = 204.3, *p* < 0.0001, Sex: *F*(1,47) = 16.4, *p* < 0.0005, Age*Sex: *F*(7,47) = 3.0.1, *p* < 0.01. *Vip-tdT+*: Age: *F*(7,52) = 222.5, *p* < 0.0001, Sex: *F*(1,52) = 36.0, *p* < 0.0001, Age*Sex: *F*(7,52) = 6.7, *p* < 0.0001. Contrasts comparing genotype or sex in each cell type, *p < 0.05.

To evaluate SCN developmental patterning, we applied these mapping approaches to samples collected from age E18-P10 (Figures 5A,B). Gestational weight, litter size, and gains in pup weight did not differ by genotype (Supplementary Figures S9A–C). Sex did not influence growth in SCN area (Supplementary Figure S2C). Overall, *Avp-tdT* mice had smaller SCN than *Vip-tdT* mice [Supplementary Figure S9D, Cre: *F*(1,84) = 8.53, *p* < 0.005], but this was only statistically significant at P05 (Supplementary Figure S9D, Contrasts, *p* < 0.01). These data indicate that the presence of *Avp-tdT* and *Vip-tdT* transgenes did not interfere with gross measures of development.

Over development, the total number of *Avp-tdT+* and *Vip-tdT+* cells increased, with differences across cell type [Figure 5C, Age: *F*(7,99) = 411.9, *p* < 0.0001, Cre: *F*(1,99) = 89.6, *p* < 0.001, Cre*Age: *F*(7,99) = 11.3, *p* < 0.0001]. In addition, sex influenced the developmental appearance of *Avp-tdT+* and *Vip-tdT+* cells [Supplementary Figure S10A, Age*Sex: *F*(7,99) = 5.17, *p* < 0.0001]. Effects of cell-type and sex persisted when cell counts were normalized to sex-specific adult values [Figure 5D, Age: *F*(7,99) = 409.4, *p* < 0.0001, Sex: *F*(1,99) = 50.18, *p* < 0.0001, Age*Sex: *F*(7,99) = 8.27, *p* < 0.0001, Cre*Age: *F*(7,99) = 2.88, *p* < 0.01], indicating that these effects were not driven by differences in the total number of cells. In each sex, a very small number of *Avp-tdT+* and *Vip-tdT+* cells were detected at E18 (Supplementary Figure S10A, *Avp* = 1.2 ± 0.1%, *Vip =* 1.9% ± 0.2% relative to adult). Population size for both cell types increased progressively after birth. When collapsed by sex, *Avp-tdT+* cells appeared between P01–P05, after which it stabilized to adult levels. In contrast, the relative number of *Vip-tdT+* cells increased from P01-P03 and P05-P10. At P05, there was a greater percentage of *Avp-tdT+* cells compared to *Vip-tdT+* cells (Figure 5C, Contrasts, *p* < 0.005). For each cell type, males displayed an accelerated appearance of *tdT*+ cells (Figure 5D). Relative to females, males had more *Avp-tdT+* cells from P01–P05 and more *Vip-tdT+* cells from P01-P10 (Figure 5D, Contrasts, *p* < 0.05). The number of labeled cells decreased to adult levels in males, and females displayed a more linear appearance of total cells for each peptide class (Figure 5D).

**Figure 6:**
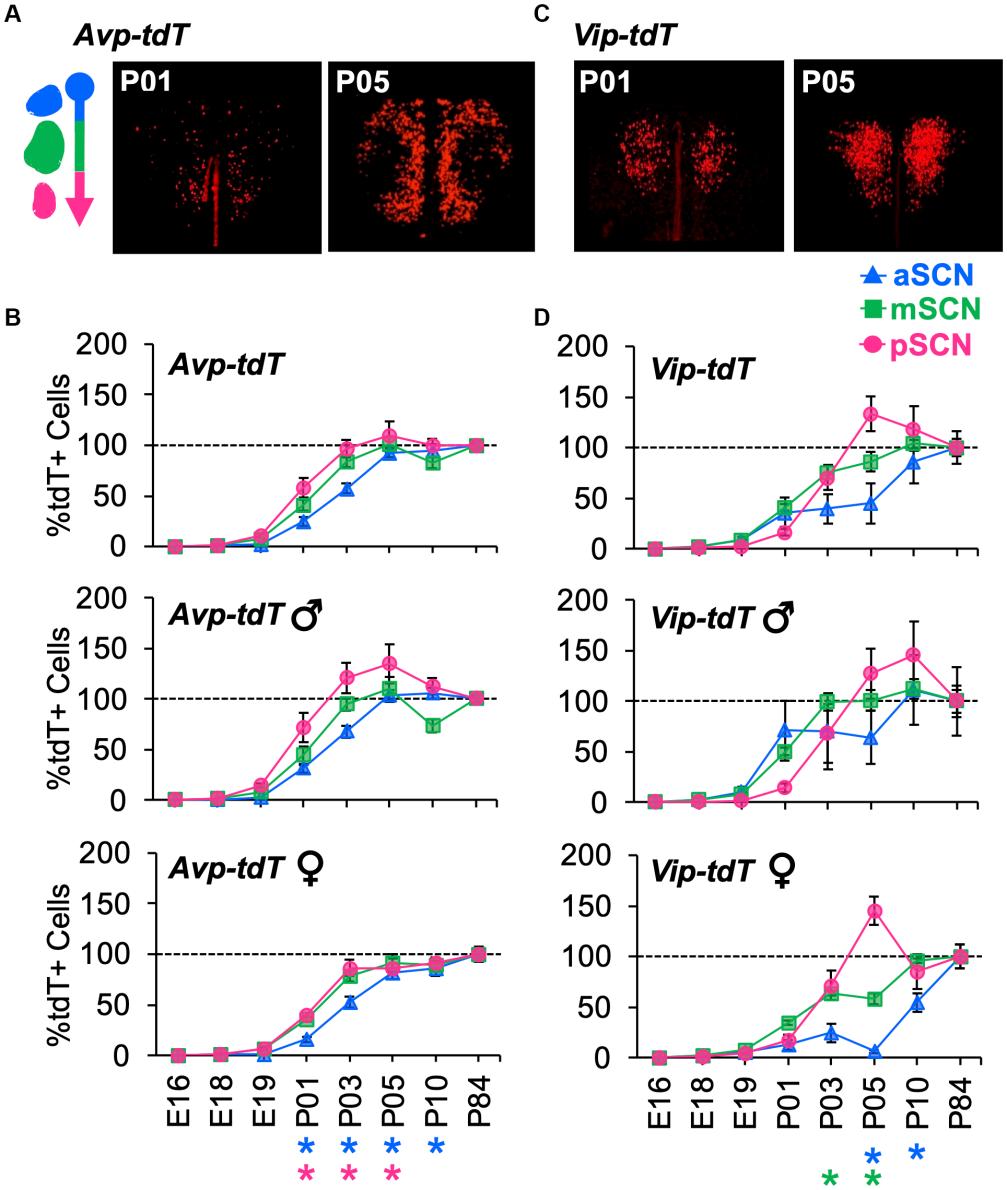
Developmental appearance of *Avp-tdT+* and *Vip-tdT+* neurons across the anteroposterior SCN. **(A)** Representative samples illustrating spatial patterning of *Avp-tdT+* development in the reconstructed horizontal plane. Coronal sections are illustrated in Supplementary Figure S10A. **(B)** Regional gradients in the developmental appearance of *Avp-tdT+* neurons are influenced by sex. Age: *F*(7,47) = 190.4, *p* < 0.0001, Sex: *F*(1,47) = 18.5, *p* < 0.0001, Position: *F*(2,94) = 14.7, *p* < 0.001, Age*Sex: *F*(7,47) = 3.3, *p* < 0.01, Age*Position: *F*(14,94) = 3.3, *p* < 0.0005, Sex*Position: *F*(2,94) = 4.2, *p* < 0.02, Age*Sex*Position: *F*(14,94) = 0.8, *p* > 0.6. **(C)** Representative samples illustrating spatial patterning *Vip-tdT+* development in the reconstructed horizontal plane. Coronal sections are illustrated in Supplementary Figure S10B. **(D)** Regional gradients in the developmental appearance of *Vip-tdT+* neurons are influenced by sex. Age: *F*(7,53) = 97.5, *p* < 0.0001, Sex: *F*(1,53) = 22.7, *p* < 0.0001, Position: *F*(2,106) = 3.3, *p* < 0.05, Age*Sex: *F*(7,53) = 4.0, *p* < 0.005, Age*Position: *F*(14,106) = 2.9, *p* < 0.001, Sex*Position: *F*(2,106) = 1.8, *p* > 0.1, Age*Sex*Position: *F*(14,106) = 0.7, *p* > 0.7. *Post hoc* contrasts comparing male and female data for each slice position are indicated by color-coded asterisks below the *x* axis of each female graph in panels **(B,D)**. *p < 0.05.

To evaluate spatial patterning, the number of cells in each class was analyzed in the anterior, middle, and posterior SCN. Age influenced cellular patterning in a manner that interacted with SCN region and sex (Figure 6, Supplementary Figure S10). Specifically, *Avp-tdT+* cells appeared in a posterior-to-anterior pattern over P01–P05, with larger regional differences in males (Figures 6A,B, Contrasts, *p* < 0.05). In the posterior SCN of males, *Avp-tdT+* cells exceeded adult levels from P03–P05 (Figure 6B). Regional patterning was also detected for *Vip-tdT+* cells, which was likewise influenced by sex (Figures 6C,D, Contrasts, *p* < 0.05). *Vip-tdT+* cells increased steadily in the middle SCN, with a larger proportion in males at P03 (Figure 6D, Contrasts, *p* < 0.05). At P05, both sexes displayed an increased proportion of *Vip-tdT+* cells in the posterior SCN that exceeded adult levels (Figure 6D). Last, the appearance of *Vip-tdT+* cells in the anterior SCN was delayed in females, with a lower percentage relative to males at P05 and P10 (Figure 6D, Contrasts, *p* < 0.05). These results suggest that there are regional gradients in the onset of peptide transcription that differ by cell type, region, and sex.

**Figure 7:**
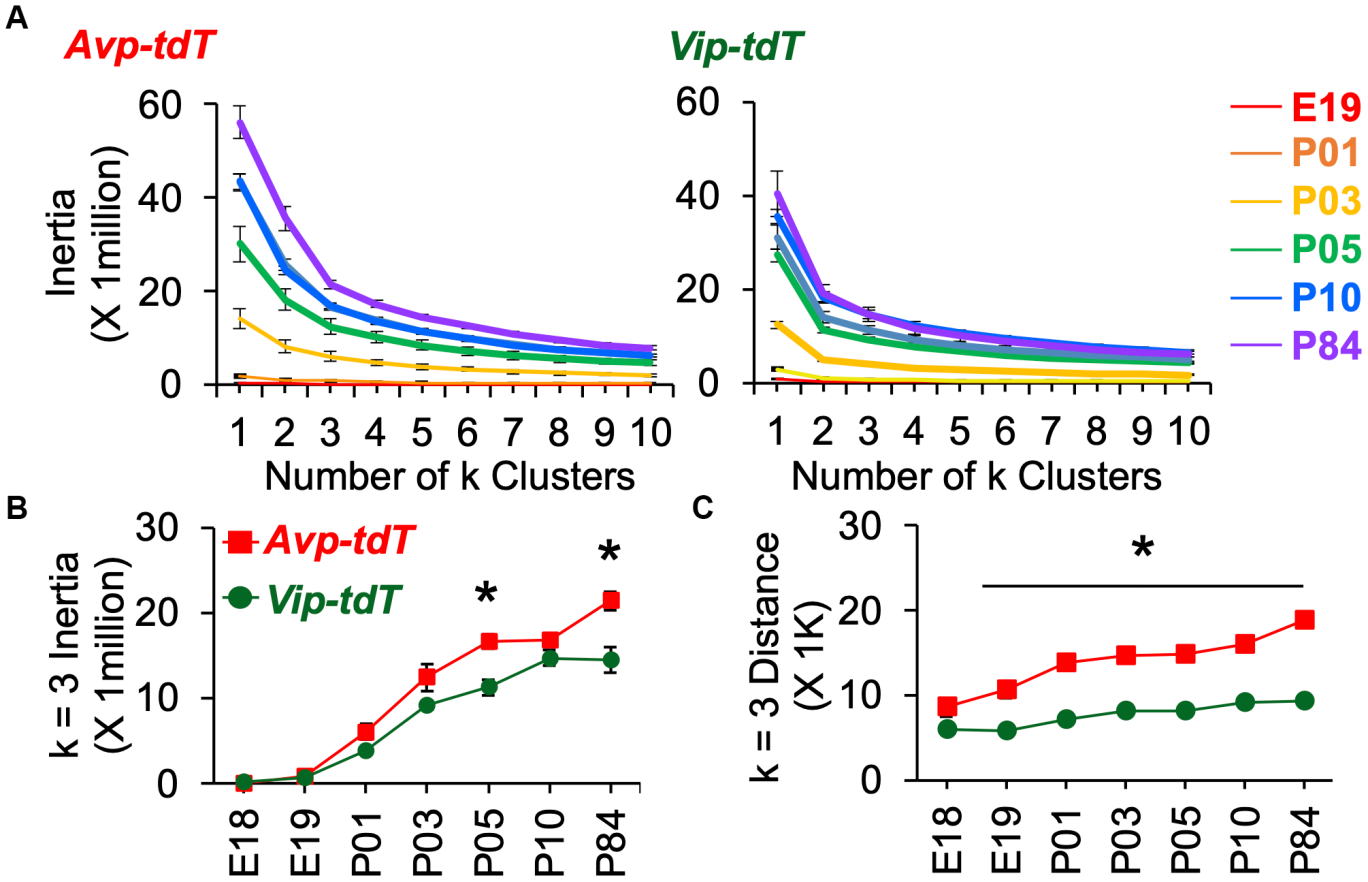
K means clustering of SCN neurons appearance across development. **(A)** Elbow plots illustrating measures of cell dispersion across age, collapsed across sex. **(B,C)** Measures of cell dispersion across age, collapsed across sex. Inertia and distance data are divided by sex in Supplementary Figure S11B. *Inertia*: Cre: *F*(1,88) = 34.4, *p* < 0.001, Age: *F*(6,88) = 124.7, *p* < 0.0001, Sex: *F*(1,88) = 0.2, *p* > 0.6, Cre*Age: *F*(6,88) = 4.0, *p* < 0.005, Cre*Sex: *F*(1,88) = 0.1, *p* > 0.7, Age*Sex: *F*(6,88) = 1.9, *p* = 0.08, Cre*Age*Sex: *F*(6,88) = 0.3, *p* > 0.9. *Distance*: Cre: *F*(1,88) = 408.4, *p* < 0.001, Age: *F*(6,88) = 33.1, *p* < 0.0001, Sex: *F*(1,88) = 2.1, *p* > 0.1, Cre*Age: *F*(6,88) = 6.7, *p* < 0.001, Cre*Sex: *F*(1,88) = 1.0, *p* > 0.3, Age*Sex: *F*(6,88) = 0.3, *p* > 0.9, Cre*Age*Sex: *F*(6,88) = 0.6, *p* > 0.7. Contrasts comparing genotype, *p < 0.05.

**Figure 8:**
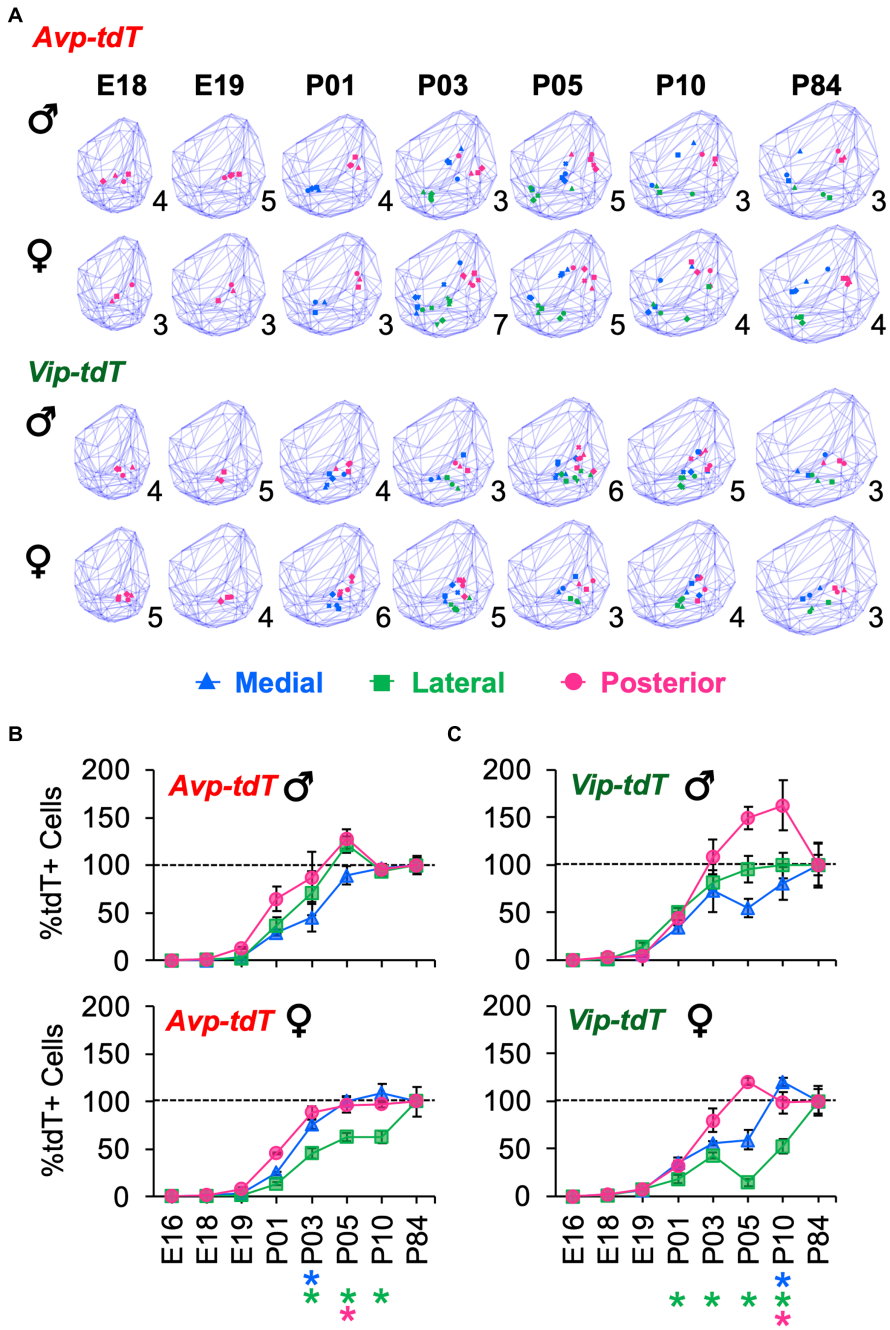
Sex differences in the development of SCN *Avp-tdT+* and *Vip-tdT+* cell clusters. **(A)** Spatial location of cellular clusters at each age for each cell type. Blue lattice frames illustrate SCN boundaries at each age. Number in bottom right corner for each map indicates number of samples. **(B,C)** Regional gradients in the developmental appearance of *Avp-tdT+* and *Vip-tdT* clusters are influenced by sex. Age: *F*(6,88) = 237.2, *p* < 0.0001, Cre: *F*(1,88) = 75.4, *p* < 0.0001, Cluster: *F*(2,176) = 144.5, *p* < 0.0001, Sex: *F*(1,88) = 0.1, *p* > 0.8, Age*Cre: *F*(6,88) = 7.4, *p* < 0.0001, Age*Cluster: *F*(12,176) = 14.7, *p* < 0.0001, Age*Sex: *F*(6,88) = 3.2, *p* < 0.01, Cre*Cluster: *F*(2,176) = 8.3, *p* < 0.0005, Cre*Sex: *F*(1,88) = 0.3, *p* > 0.6, Cluster*Sex: *F*(2,176) = 5.1, *p* < 0.01, Age*Cre*Cluster: (12,176) = 4.4, *p* < 0.0001, Age*Cluster*Sex: *F*(12,176) = 1.9, *p* < 0.05, Age*Cre*Cluster*Sex: *F*(12,176) = 1.5, *p* > 0.1. *Post hoc* contrasts comparing male and female data for each slice position are indicated by color-coded asterisks below the *x* axis of each female graph in Figures 6B,C. *p < 0.05.

**Figure 9:**
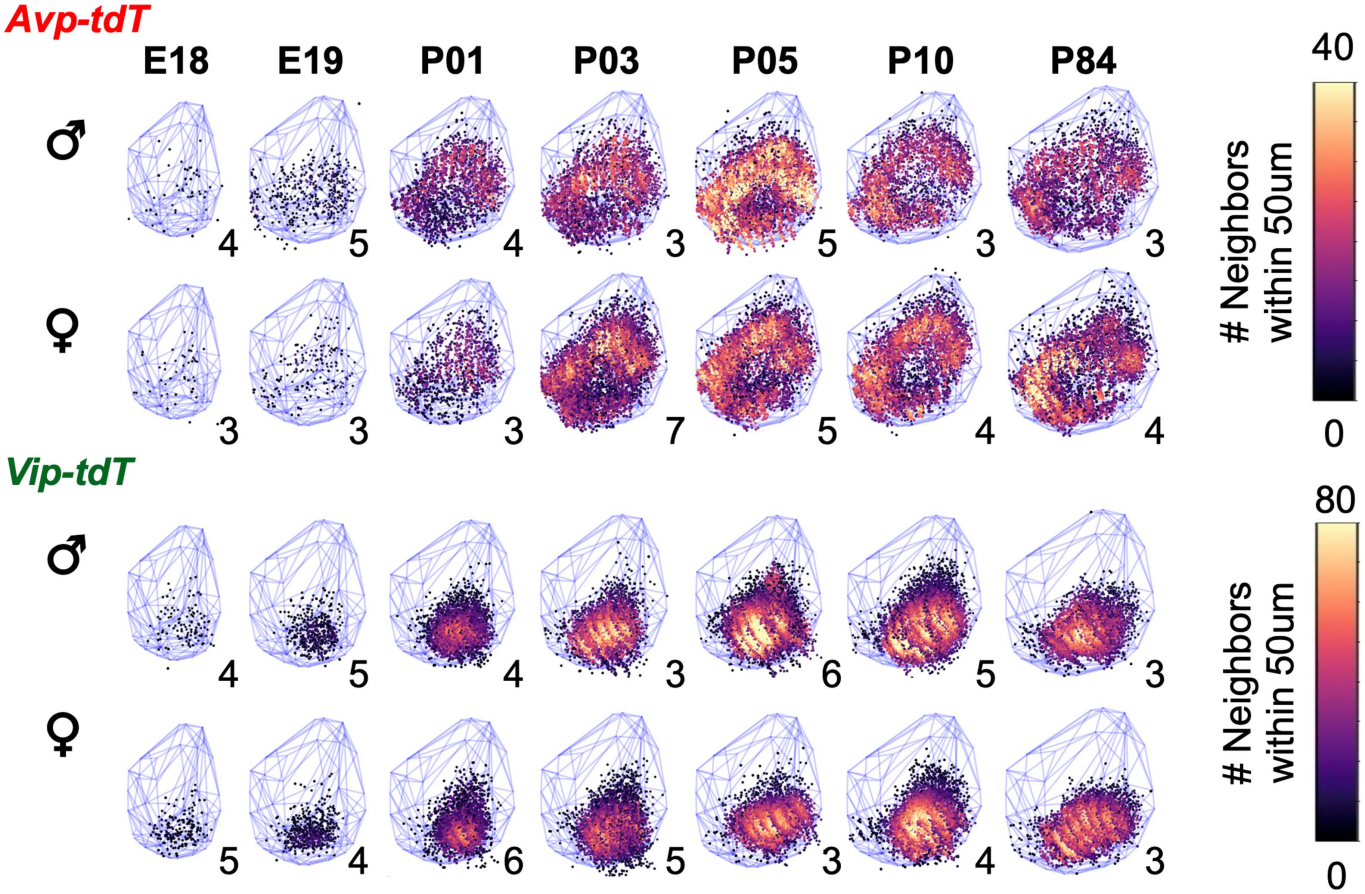
Sex differences in the developmental patterning of SCN *Vip-tdT* cell density plots aggregated across samples for each age illustrating total number of neighboring cells for each cell type in each sex. Blue lattice frames illustrate SCN boundaries at each age. Number in bottom right corner for each map indicates the number of aggregated samples. Additional orientations at select ages are shown in Supplementary Videos S1–S12.

For each cell type, cell dispersion within k-means clusters increased as the SCN grew with age (Figure 7A). Cellular dispersion was greater in *Avp-tdT+* than *Vip-tdT+* cells (Figures 7B,C, Supplementary Figure S11B, Contrasts, *p* < 0.05). *Avp-tdT+* cells displayed a stepwise pattern of increasing cell dispersion over P03–P10 (Figure 7A), likely since this cell type spans the anteroposterior extent of the SCN. Developmental patterning of cellular appearance and density was influenced by sex (Figures 8, 9). The spatial location of clusters was largely similar in each sex across development (Figure 8A), but sex influenced the appearance of cells in different clusters (Figures 8B,C). In male SCN, there was a greater number of *Avp-tdT+* cells in the posterior cluster at P05 and in the lateral cluster over P03–P10 relative to females (Figure 8B, Contrasts, *p* < 0.05). In addition, males had more *Vip-tdT+* cells in the lateral cluster from P01–P10 and in the posterior cluster at P10 (Figure 8C, Contrasts, *p* < 0.05). In contrast, females displayed more *Avp-tdT+* cells in the medial cluster at P03 and more *Vip-tdT+* cells in the medial cluster at P10 (Figures 8B,C, Contrasts, *p* < 0.05). Overall, male SCN displayed higher cell density for *Avp-tdT+* and *Vip-tdT+* cells at P05 relative to females (Figure 9). Collectively, these results indicate that the developmental patterning of SCN *Avp-tdT+* and *Vip-tdT+* cells differs by sex.

## Discussion

Hypothalamus anatomy is conserved across vertebrates, guided by molecular mechanisms that determine nuclei that contain a large diversity of neuron subtypes (Xie and Dorsky, 2017; Benevento et al., 2022). Relative to early induction, less is known about how these peptide circuits are built and remodeled. In the SCN, AVP and VIP neurons regulate the timing of sleep, stress, and reproductive rhythms. How peptide circuits in the SCN network are patterned over development may have profound impacts on clock function in adulthood. Using a genetic approach to track SCN development of peptide circuits, our results suggest that SCN patterning varies by cell type, regional subcluster, and sex. The genetic and/or hormonal factors that guide spatial patterning of SCN peptide circuits warrant further research.

Genetic labeling provides insight into cellular appearance over development without the need for surgical or chemical interventions that could interfere with gestation and rearing, but this approach is not without limitations. Both *Avp-tdT* and *Vip-tdT* expression were Cre-dependent consistent with previous work describing these genetic models (Harris et al., 2014; Taniguchi, 2014). We find that 70% of *Avp-tdT+* neurons were AVP-IHC+, but less than 30% of *Vip-tdT+* neurons were VIP-IHC+. Low VIP co-expression could reflect transient *Vip* transcription over development in a large subset of these cells, which would suggest that the VIP cell population may expand and contract over development. However, this observation could also reflect threshold limits of IHC and/or expression of VIP-related peptides that are not recognized by the antibody used here (Lee et al., 2013; Southey et al., 2014). On the other hand, over 80% of VIP+ cells were labeled with *Vip-tdT*, but only 43% of AVP+ cells were labeled by *Avp-tdT*. This has been noted in previous work using this genetic model (Jamieson, 2020), and our data indicate that the dorsal region of the middle SCN displays the highest rate of AVP and *Avp-tdT* discordance. Failure of Cre-mediated recombination may reflect cellular variation in epigenetic landscape or genetic history (i.e., loss of Cre during cell division), as suggested in previous work (Jamieson, 2020). Interestingly, low co-expression of AVP and *Avp-tdT* labeling also occurs in this genetic model after adult-specific viral transduction (Jamieson, 2020), which suggests that this observation is not a developmental artifact. Further, we used colchicine to visualize total protein expression over the daily cycle, yet our estimates of colocalization are similar to this previous work (Jamieson, 2020). Collectively, these data indicate that each mouse model used here does not fully capture peptide expression in adulthood, thus limiting the ability to comprehensively map each peptide population during development. However, these validation data also provide an interesting complement to our developmental results by suggesting that neurons in each peptide class may be divided into subclusters. Another known caveat of Cre models is that the transgene can interfere with native peptide expression (Cheng et al., 2019; Joye et al., 2020; Rohr et al., 2020). Importantly, peptide levels in heterozygous *Avp-tdT* and *Vip-tdT* mice do not differ from wildtype mice during early development, and circadian behavior does not differ between these two groups during adulthood (Joye et al., 2020; Rohr et al., 2020). However, it is difficult to dismiss that a non-significant decrease in peptide expression could alter SCN patterning. With these caveats in mind, we decided to employ this genetic approach to study SCN peptide development because it avoids the need to conduct a circadian time course at every age.

Consistent with previous work, we find developmental differences in the appearance of SCN neurons in these two peptide classes. *Avp-tdT+* and *Vip-tdT+* neurons were detected as early as E18, and cell number for each peptide class increased over the first 10 days after birth. Our results align well with previous work characterizing peptide development, despite the likely delays between transcription onset and tdT labeling. *Avp* and *Vip* transcripts are first detected in the mouse SCN at E17-18 and E18-19, respectively (Vandunk et al., 2011). AVP and VIP peptide levels increase over the first 2 days after birth (Hyodo et al., 1992; Carmona-Alcocer et al., 2018). SCN AVP cell numbers are stable after P06, but AVP peptide levels continue to increase from P06-P30 (Herzog et al., 2000), which would not be captured with the present approach. Interesting, VIP cell numbers increase between P06 and P30 (Herzog et al., 2000), which we also observed in the present work. It remains unclear what molecular factors drive ontogenetic patterning in these two different SCN peptide classes. The expression of transcription factors during embryogenesis (e.g., Lhx1, Shh, Six3, Six6) is important in early SCN specification (Bedont and Blackshaw, 2015; Carmona-Alcocer et al., 2020). Lhx1 and Foxd1 deletion decreases both *Avp* and *Vip* expression (Vandunk et al., 2011; Newman et al., 2018), suggesting common genetic programs direct cellular differentiation in both classes. Differences in the timing of developmental patterning across these two cell types may be linked to intrinsic and/or extrinsic factors. Over the first week of life, terminal differentiation, synaptogenesis, gliogenesis, and retinal innervation occurs in the SCN (Bedont and Blackshaw, 2015; Carmona-Alcocer et al., 2020), and later maturation of the VIP population may be linked to postnatal maturation of retinal inputs (McNeill et al., 2011). Both AVP and VIP influence SCN circuit formation and function during development (Ono et al., 2016; Bedont et al., 2018; Mazuski et al., 2020); thus, the timing and patterning of these peptides may have important consequences for pacemaker function.

Notably, we find that SCN peptide classes can be further subdivided based on spatial patterning during development. For *Avp-tdT+* neurons, we find that there is a posterior–anterior gradient when analyzed by anatomical division based on slice position. Consistent with these results, k means clustering detected that the posterior *Avp-tdT+* cluster matured faster than other clusters in each sex, with development of one of the more anterior *Avp-tdT+* clusters delayed in a sex-influenced manner. For *Vip-tdT+* neurons, we observed a rapid increase of cells in the posterior cluster. Consistent with this result, VIP neurons have been reported to increase in the middle and posterior SCN over P06 to P30 in the mouse SCN (Herzog et al., 2000). In rats, two developmental waves of *Vip* expression have been reported, with *Vip* transcription occurring in medial SCN cells earlier than lateral SCN cells (Ban et al., 1997; Kawamoto et al., 2003). These two spatially defined subclusters displayed different patterns of clock gene expression and photic sensitivity in adulthood (Ban et al., 1997; Kawamoto et al., 2003). Further, VIP neurons in adulthood can be divided into two subpopulations based on electrical firing (Mazuski et al., 2018) and *Grp* expression (Todd et al., 2020; Wen et al., 2020). Interestingly, recent work in the mouse has found two subsets of *Avp* cells that differ in the expression of *Cck* or *Nms* (Moffitt et al., 2018). Whether functional subclasses of SCN *Vip* and *Avp* cells map onto the regional subclusters found here would be interesting to examine in future work. It is also unclear how the current spatial gradients may relate to those found for SCN neurogenesis (Okamura et al., 1983; Kabrita and Davis, 2008). In the mouse, SCN neurogenesis occurs over embryonic days 11–16 (E11–16), with a peak at E14 (Shimada and Nakamura, 1973; Kabrita and Davis, 2008). SCN core neurons are generated at an earlier age (peak at E12) than shell neurons (peak E13.5) in the mouse (Kabrita and Davis, 2008). In the hamster, AVP neurons are generated over a longer period of gestation than VIP and GRP neurons in the SCN core, with posterior-to-anterior gradients (Antle et al., 2005). The degree to which onset of neuropeptide expression is timed by neurogenesis and/or extrinsic cues present in the microenvironment (Xie and Dorsky, 2017; Benevento et al., 2022) remains an open question.

Last, our results suggest that developmental patterning of peptide circuits is influenced by sex. The male SCN displayed postnatal increases in *Avp-tdT+* and *Vip-tdT+* cell number and density that were not maintained into adulthood. The number of *Vip-tdT+* cells at P05–P10 exceeded adult levels in male SCN by 20%, and the number of *Avp-tdT+* neurons at P05 exceeded adult levels in male SCN by 14%. Given that tdT labeling is permanent, this observation suggests a loss of SCN cells in males. The majority of SCN apoptosis occurs over P01-P07 in mice, but an estimated 20% of cells are lost between P07 and adulthood (Ahern et al., 2013; Bedont et al., 2014; Mosley et al., 2017). In contrast, the female SCN displayed a more linear patterning of *Avp-tdT+* and *Vip-tdT+* cell appearance, with increasing numbers of both cell types between P10 and P84. In addition, the more anterior *Avp-tdT+* cluster that matured last differed by sex, with the lateral cluster appearing last in females and the medial cluster appearing last in males. Interestingly, the lateral cluster had significantly more *Avp-tdT+* neurons in adult females than males due to the increase in cell number after P10. Likewise, the *Vip-tdT+* cluster that matured last differed by sex, again being the lateral cluster in females and the medial cluster in males. Cell number in the lateral *Vip-tdT+* cluster also differed by sex due to post-P10 increases in cell numbers in females. Collectively, these data indicate that SCN development is not complete by P10, raising the possibility that puberty represents another time of SCN development (Bakker and Baum, 2008).

Whether these sex differences are driven by genetic and/or hormonal factors remains to be tested, but it is tempting to speculate that sex steroids are organizing development of SCN circuits. The critical period in the mouse is E18-P01, with testosterone levels decreasing rapidly at birth and the sensitive period in females extending to P07 (McCarthy et al., 2018). The mouse SCN expresses receptors for sex steroids that are regionally clustered in adulthood (Joye and Evans, 2021), potentially contributing to the spatial gradients in peptide development observed here. Sex differences in SCN neurogenesis have been reported in mice (Abizaid et al., 2004). Specifically, females display more SCN neurogenesis at E18, and testosterone administration to pregnant dams reduces SCN neurogenesis at this late stage of gestation (Abizaid et al., 2004). This suggests that neurogenesis closes earlier in males (Shimada and Nakamura, 1973; Kabrita and Davis, 2008) due to sex steroid signaling. Last, the peak in SCN apoptosis occurs at P03 in males and P05 in females with equivalent postnatal SCN volume (Ahern et al., 2013). To our knowledge, sex steroid receptor expression over early SCN development has not been examined in the mouse, but androgen receptors are expressed later in life in the slow-maturing diurnal rodent *Octogon degus* (Lee et al., 2004). Overall, our work suggests that SCN shape and peptide expression is influenced by sex, as reported in humans (Swaab et al., 1985, 1994). Future work is warranted to further explore how adult clock function in both sexes is influenced by SCN patterning during development and how this process is impacted by postnatal conditions, such as light exposure (Cambras et al., 1998, 2015; Ohta et al., 2006; Ciarleglio et al., 2011; Ono et al., 2013; Madahi et al., 2018).

A central question here concerned the spatial patterning of SCN maturation, which we have represented in 3D maps for two different peptide classes at critical developmental time points spanning mid-embryonic age to adulthood in both sexes. Both AVP and VIP neurons provide local and long-range cues that regulate daily rhythms (Vosko et al., 2007; Kalsbeek et al., 2010; Mieda et al., 2016; Rohr et al., 2020; Shan et al., 2020). In addition to shell-core compartmentalization, cellular differences in clock function are also evident across the anteroposterior axis (Hamada et al., 2004; Yan et al., 2007; Evans et al., 2011; Yoshikawa et al., 2015, 2017). Do regional and sex differences in peptide development relate to differences in cellular clock function in adulthood? Does the spatial patterning of the SCN circuit extend to other developmental milestones (e.g., gliogenesis, axonal projections)? Do sexual dimorphisms in spatial patterning relate to sex differences in peptide expression and clock function in adulthood? More broadly, how do changes in the conditions present during the postnatal SCN developmental period influence circuit formation to modulate adult clock function? Future work investigating these questions may provide insight into the long-lasting effects of perinatal light exposure on health (Torrey et al., 2000; Madahi et al., 2018).

## Data availability statement

The raw data supporting the conclusions of this article will be made available by the authors, without undue reservation.

## Ethics statement

The animal study was reviewed and approved by the Institutional Animal Care and Use Committees at Marquette University.

## Author contributions

JAE wrote the manuscript with contributions and approval of submitted version by all authors. All authors contributed to experimental design, performed research and analyzed data.

## Funding

This work was supported by the National Institutes of Health, R01GM143545.

## Conflict of interest

The authors declare that the research was conducted in the absence of any commercial or financial relationships that could be construed as a potential conflict of interest.

## Publisher’s note

All claims expressed in this article are solely those of the authors and do not necessarily represent those of their affiliated organizations, or those of the publisher, the editors and the reviewers. Any product that may be evaluated in this article, or claim that may be made by its manufacturer, is not guaranteed or endorsed by the publisher.
