# Supplementary material for: Developmental patterning of peptide transcription in the central circadian clock in both sexes
Source: Front Neurosci. 2023 May 19;17:1177458. doi: 10.3389/fnins.2023.1177458 (PMC10235759; doi:10.3389/fnins.2023.1177458)
Supplement: Supplementary file 1 [file Data_Sheet_1.PDF]

Supplemental Materials for:

Developmental patterning of peptide transcription in the central circadian clock in both sexes

Vania Carmona-Alcocer, Lindsey S. Brown,
Aiesha Anchan, Kayla E. Rohr, Jennifer A. Evans

Supplemental Figure Legends

Figure S1. Spatial mapping of SCN *Avp-tdT*+ and *Vip-tdT*+ neurons. (A) Representative images illustrating *Avp-tdT*+ and *Vip-tdT*+ neurons across the anteroposterior SCN. Note each slice is color-coded by position and superimposed in rightmost panel using the hyperstack temporal color code. (B) Cells expressing *Avp-tdT* and *Vip-tdT*, divided by anterior, middle, and posterior SCN for representative mice of each sex. (C) Cells expressing *Avp-tdT* and *Vip-tdT* divided by anterior, middle, and posterior SCN for all mice of each sex (top, middle) or all mice collapsed by sex (bottom).

Figure S2. SCN slice atlas over development. (A) Representative slice sets for SCN collected at each age. The slices used for anterior, middle and posterior SCN are color coded by age. (B) DAPI-derived boundaries of the anterior, middle, and posterior SCN across development. (C) SCN area divided by sex across age Cre: $F(1,84) = 8.53$, $p < 0.005$, Age: $F(6,84) = 59.32$, $p < 0.0001$, Sex: $F(1,84) = 1.08$, $p > 0.3$, Cre*Age: $F(6,84) = 1.2$, $p > 0.3$, Cre*Sex: $F(1,84) = 0.0001$, $p > 0.9$, Age*Sex: $F(1,84) = 0.66$, $p > 0.6$, Cre*Age*Sex: $F(6,84) = 0.93$, $p > 0.4$.

Figure S3. Mapping SCN cell density. (A) Representative cell density maps for an individual image illustrating cell density calculated by counting the number of neighboring cells in a 10, 50, or 100 μm radius. (B) Front and lateral view of *Avp-tdT*+ cell density maps illustrating number of cells within a 50 μm radius for each individual sample. (C) Front and lateral view of *Vip-tdT*+ cell density maps illustrating number of cells within a 50 μm radius for each individual sample.

Figure S4. SCN *Avp-tdT* and *Vip-tdT* labeling is Cre dependent. (A) Representative images illustrating tdT expression in each genotype. (B-C) Total number of SCN tdT+ cells in each genotype (B) and divided by slice position (C). Cre: $F(2,22) = 24$, $p < 0.0001$, Sex: $F(1,22) = 0.3$, $p > 0.6$, Position: $F(2,44) = 2.9$, $p = 0.07$, Cre*Sex: $F(2,22) = 0.6$, $p > 0.5$, Cre*Position: $F(4,44) = 8.1$, $p < 0.0001$, Sex*Position: $F(2,44) = 0.2$, $p > 0.8$, Cre*Sex*Position: $F(4,44) = 0.1$, $p > 0.9$. (D) tdT expression in the cortex. $t(7) = 8.39$, $p < 0.0005$. Scale bars = 100 μm.

Figure S5. Evaluation of genetic strategy to label spatial location of SCN peptide classes. (A-B) Anterior, middle, and posterior SCN illustrating tdT and AVP/VIP expression in *Avp-tdT* and *Vip-tdT* samples collected from each sex. (C-D) Number of SCN neurons labeled by tdT or AVP/VIP immunohistochemistry (IHC) in each genotype and sex. **AVP/VIP IHC**: Cre: $F(1,15) = 175$, $p < 0.0001$; Sex: $F(1,15) = 0.2$, $p > 0.6$; Cre*Sex: $F(1,15) = 0.3$, $p > 0.5$. **tdT+:** Cre: $F(1,15) = 4.12$, $p = 0.06$; Sex: $F(1,15) = 2.3$, $p > 0.1$; Cre*Sex: $F(1,15) = 2.9$, $p > 0.1$. (E-F) Co-expression of tdT and AVP/VIP labeling does not differ by sex. **%tdT+AVP-IHC-** Sex: $F(1,8) = 1.2$, $p > 0.3$; Position: $F(2,16) = 2.7$, $p = 0.09$; Sex*Position: $F(2,16) = 0.4$, $p > 0.6$. **%tdT+VIP-IHC-** Sex: $F(1,7) = 9.7$, $p = 0.02$; Position: $F(2,13) = 0.7$, $p > 0.5$; Sex*Position: $F(2,13) = 1.1$, $p > 0.3$. **%AVP-IHC+tdT-** Sex: $F(1,8) = 4.2$, $p = 0.08$; Position: $F(2,16) = 7.9$, $p < 0.005$; Sex*Position: $F(2,16) = 0.2$, $p > 0.8$. **%VIP-IHC+tdT-** Sex: $F(1,7) = 1.2$, $p > 0.3$; Position: $F(2,13) = 2.2$, $p > 0.1$; Sex*Position: $F(2,13) = 0.3$, $p > 0.7$. Scale bars = 100 μm.

Figure S6. K means clustering on cellular dispersion in each SCN peptide class. (A) Elbow plots illustrating measures of cellular dispersion without and with normalization to total SCN cells of each sample (i.e., Inertia, Distance), divided by sex. (B) There was greater cellular dispersion in *Avp-tdT+* neurons than *Vip-tdT+* neurons in each sex. **Inertia** – Cre: $F(1,9) = 17.5$, $p < 0.005$, Sex: $F(1,9) = 4.8$, $p = 0.06$; Sex*Cre: $F(1,9) = 0.001$, $p > 0.9$. **Distance**– Cre: $F(1,9) = 99.2$, $p < 0.0001$, Sex: $F(1,9) = 0.12$, $p > 0.7$; Sex*Cre: $F(1,9) = 0.02$, $p > 0.8$.

Figure S7. Cell density maps of *Avp-tdT+* neurons in the adult SCN. (A) Cell density maps illustrating the number of neighboring cells for all mice of each sex. (B) Cell density maps illustrating the number of neighboring cells in each sex relative to the total number of labeled neurons in each sample.

Figure S8. Cell density of *Vip-tdT+* neurons in the adult SCN. (A) Cell density maps illustrating the number of neighboring cells for all mice of each sex. (B) Cell density maps illustrating the number of neighboring cells in each sex relative to the total number of labeled neurons in each sample.

Figure S9. Pregnancy and pup weight does not differ by offspring genotype. (A) Maternal weight gain in females pregnant with pups of each genotype. $F(2,38) = 0.15$, $p > 0.8$. (B) Litter size in dams bearing pups of each genotype $F(2,38) = 0.1$, $p > 0.9$. (C) Pup weight over development did not differ by genotype. Genotype: $F(2,18) = 0.76$, $p > 0.4$; Age: $F(3,18) = 157.3$, $p < 0.001$; Genotype*Age: $F(6,18) = 0.38$, $p > 0.8$. D. SCN size in pups of each genotype. Genotype: $F(1,84) = 8.53$, $p < 0.005$, Age: $F(6,84) = 59.32$, $p < 0.0001$, Sex: $F(1,84) = 1.08$, $p > 0.3$, Cre*Age: $F(6,84) = 1.2$, $p > 0.3$, Cre*Sex: $F(1,84) = 0.0001$, $p > 0.9$, Age*Sex: $F(6,84) = 0.66$, $p > 0.6$, Cre*Age*Sex: $F(6,84) = 0.93$, $p > 0.4$. n.s. not significant. * $p < 0.05$.

Figure S10. Anteroposterior differences in the appearance of SCN *Avp-tdT+* and *Vip-tdT+* cells over postnatal development. (A-B) Representative coronal images of labeled cells in each SCN peptide class across postnatal ages, summed across the anterior, middle, or posterior SCN.

Figure S11. Developmental appearance of total labeled cells (A) and measures of cell dispersion (B) divided by sex. (A) Cre: $F(1,99) = 89.6$, $p < 0.001$, Age: $F(7,99) = 411.9$, $p < 0.0001$, Sex: $F(1,99) = 0.7$, $p > 0.4$, Cre*Age: $F(7,99) = 11.25$, $p < 0.0001$, Cre*Sex: $F(1,99) = 0.01$, $p > 0.9$, Age*Sex: $F(7,99) = 5.2$, $p < 0.0001$, Cre*Age*Sex: $F(7,99) = 0.8$, $p > 0.5$. (B) **Inertia:** Cre: $F(1,88) = 34.4$, $p < 0.001$, Age: $F(6,88) = 124.7$, $p < 0.0001$, Sex: $F(1,88) = 0.2$, $p > 0.6$, Cre*Age: $F(6,88) = 4.0$, $p < 0.005$, Cre*Sex: $F(1,88) = 0.1$, $p > 0.7$, Age*Sex: $F(6,88) = 1.9$, $p = 0.08$, Cre*Age*Sex: $F(6,88) = 0.3$, $p > 0.9$. **Distance:** Cre: $F(1,88) = 408.4$, $p < 0.001$, Age: $F(6,88) = 33.1$, $p < 0.0001$, Sex: $F(1,88) = 2.1$, $p > 0.1$, Cre*Age: $F(6,88) = 6.7$, $p < 0.001$, Cre*Sex: $F(1,88) = 1.0$, $p > 0.3$, Age*Sex: $F(6,88) = 0.3$, $p > 0.9$, Cre*Age*Sex: $F(6,88) = 0.6$, $p > 0.7$.

Supplementary Videos

Video S1. *Avp-tdT+* cell density in adult P84 males. Rotating view of adult male SCN. *Avp-tdT+* neurons are collapsed across samples. Color represents number of neighboring cells in a 50 μm radius.

Video S2. *Vip-tdT+* cell density in adult P84 males. Conventions as in Video S1.

Video S3. *Avp-tdT+* cell density in adult P84 females. Conventions as in Video S1.

Video S4. *Vip-tdT+* cell density in adult P84 females. Conventions as in Video S1.

Video S5. ***Avp-tdT*****+ cell density in P01 males.** Conventions as in Video S1.

Video S6. ***Avp-tdT*****+ cell density in P05 males.** Conventions as in Video S1.

Video S7. ***Avp-tdT*****+ cell density in P01 females.** Conventions as in Video S1.

Video S8. ***Avp-tdT*****+ cell density in P05 females.** Conventions as in Video S1.

Video S9. ***Vip-tdT*****+ cell density in P01 males.** Conventions as in Video S1.

Video S10. ***Vip-tdT*****+ cell density in P05 males.** Conventions as in Video S1.

Video S11. ***Vip-tdT*****+ cell density in P01 females.** Conventions as in Video S1.

Video S12. ***Vip-tdT*****+ cell density in P05 females.** Conventions as in Video S1.

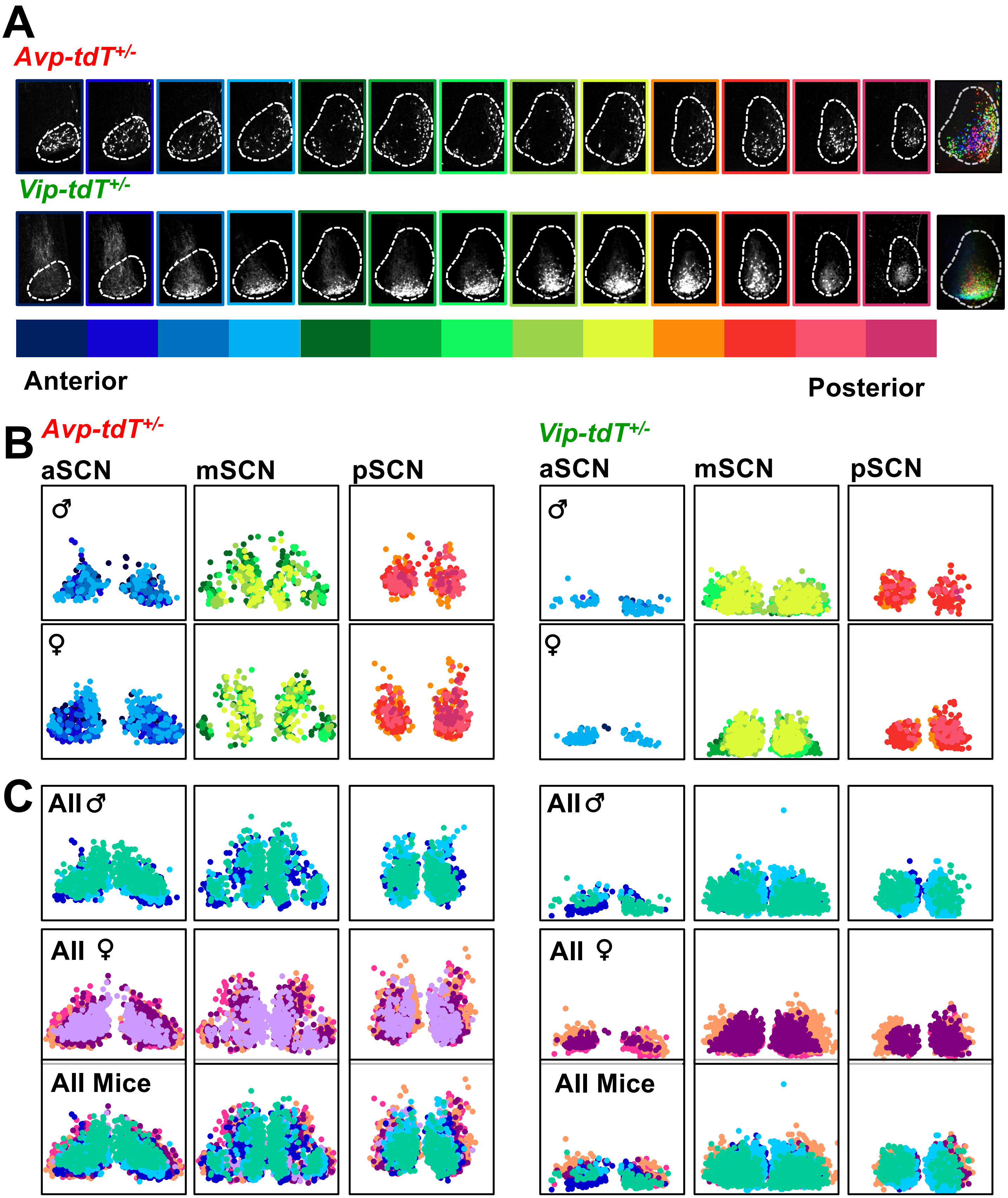
A
Avp-tdT+/-
Vip-tdT+/-
Anterior
Posterior
B
Avp-tdT+/-
aSCN
mSCN
pSCN
♂
♀
Vip-tdT+/-
aSCN
mSCN
pSCN
♂
♀
C
All ♂
All ♀
All Mice
All ♂
All ♀
All Mice

Figure S1

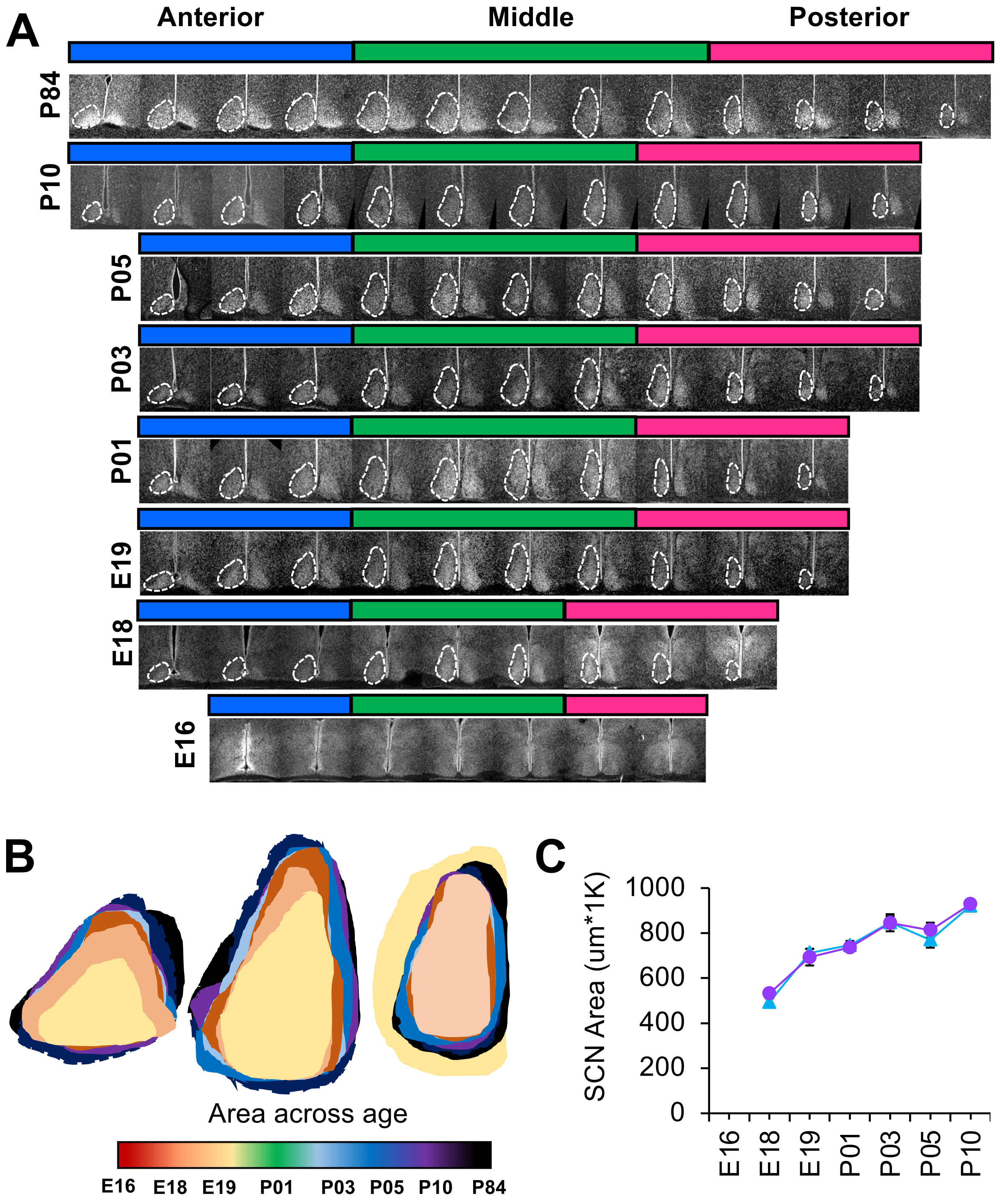

A
Anterior
Middle
Posterior
P84
P10
P05
P03
P01
E19
E18
E16
B
Area across age
E16
E18
E19
P01
P03
P05
P10
P84
C
SCN Area (um*1K)
1000
800
600
400
200
0
E16
E18
E19
P01
P03
P05
P10

Figure S2

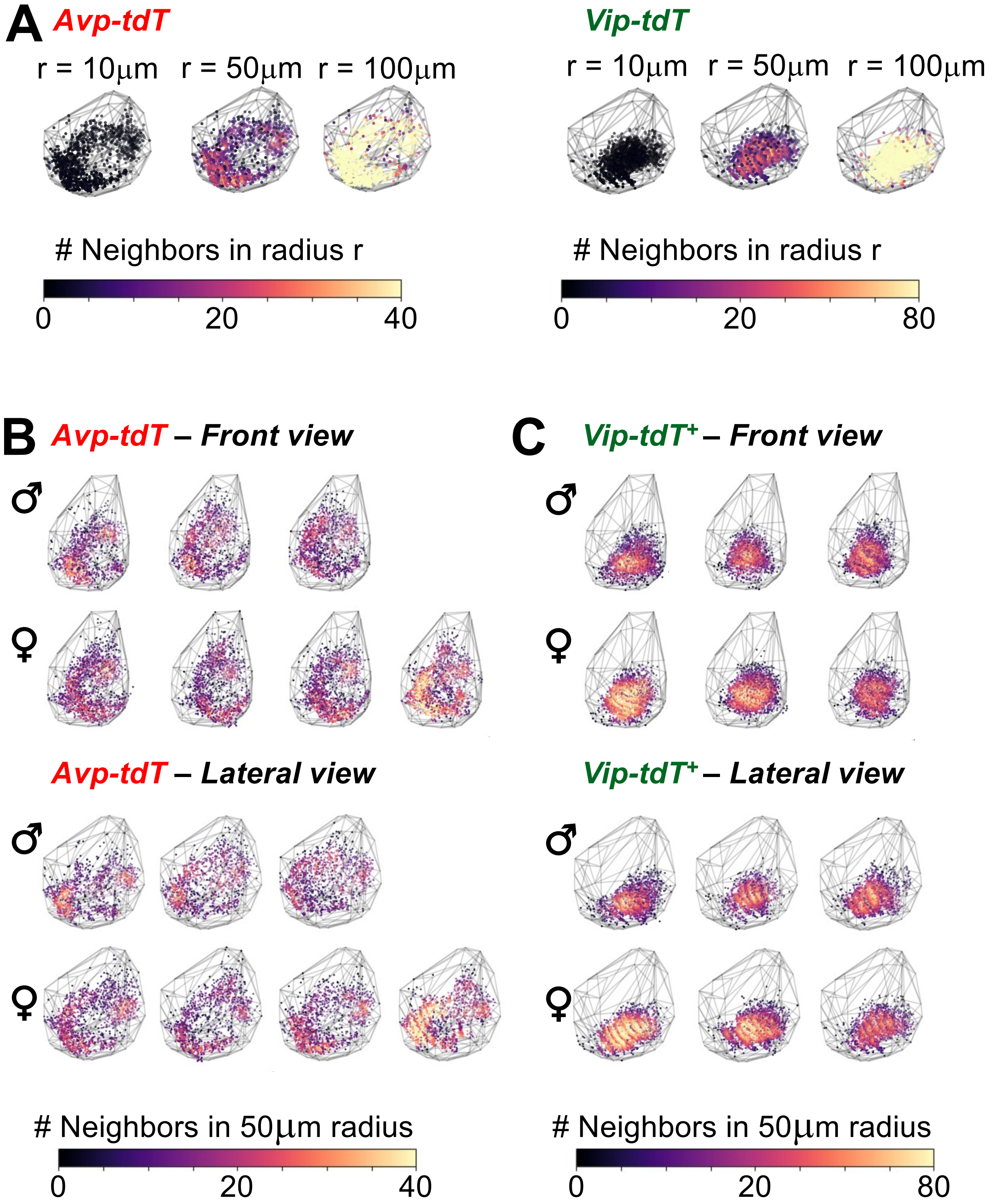

A Avp-tdT
r = 10μm r = 50μm r = 100μm
# Neighbors in radius r
0 20 40
Vip-tdT
r = 10μm r = 50μm r = 100μm
# Neighbors in radius r
0 20 80
B Avp-tdT – Front view
♂
♀
Avp-tdT – Lateral view
♂
♀
Neighbors in 50μm radius
0 20 40
C Vip-tdT+ – Front view
♂
♀
Vip-tdT+ – Lateral view
♂
♀
Neighbors in 50μm radius
0 20 80

Figure S3

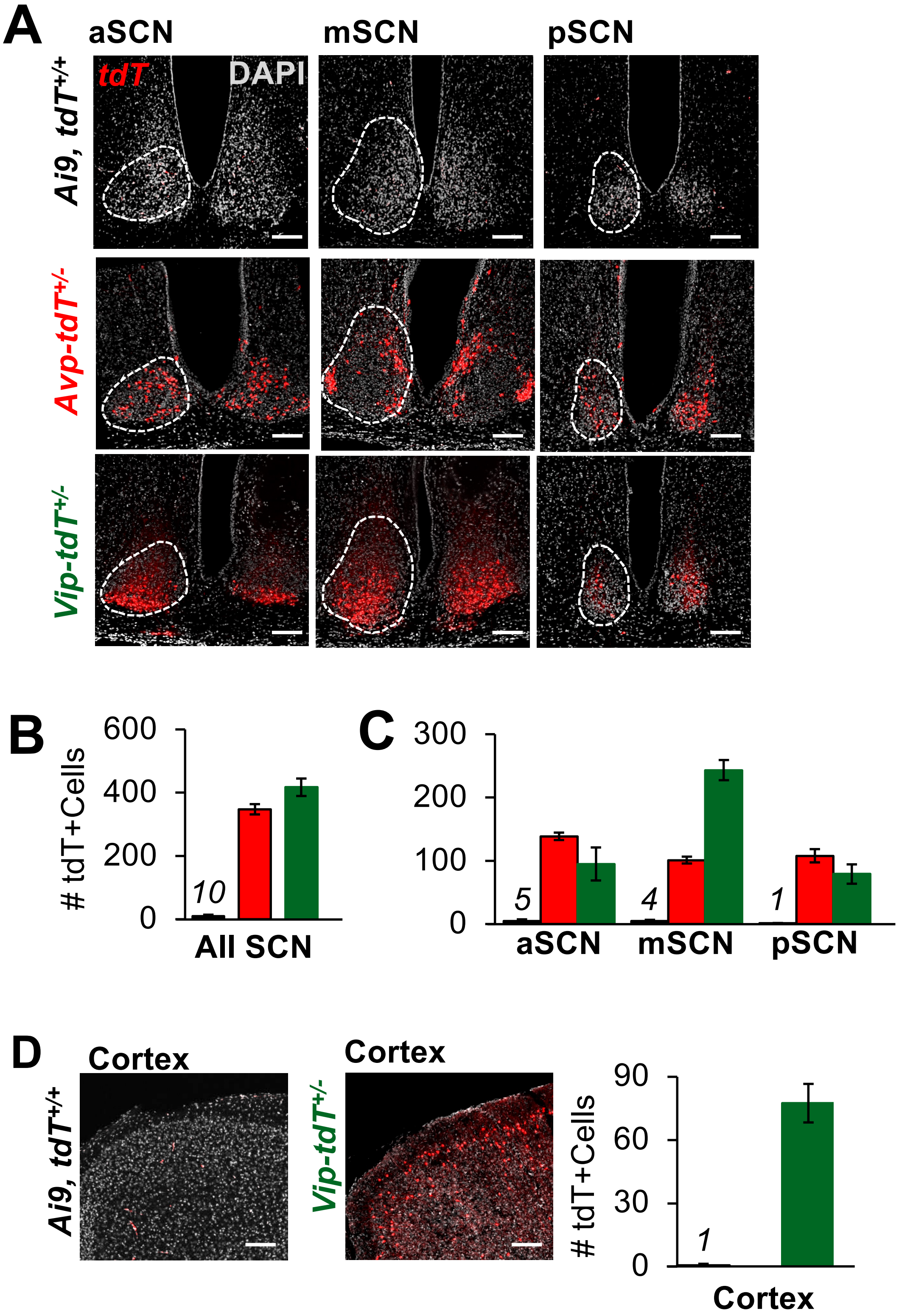
A
aSCN
mSCN
pSCN
tdT
DAPI
Ai9, tdT+/+
Avp-tdT+/-
Vip-tdT+/-
B
tdT+Cells
600
400
200
0
10
All SCN
C
300
200
100
0
5
4
1
aSCN
mSCN
pSCN
D
Cortex
Ai9, tdT+/+
Cortex
Vip-tdT+/-
tdT+Cells
90
60
30
0
1
Cortex

Figure S4

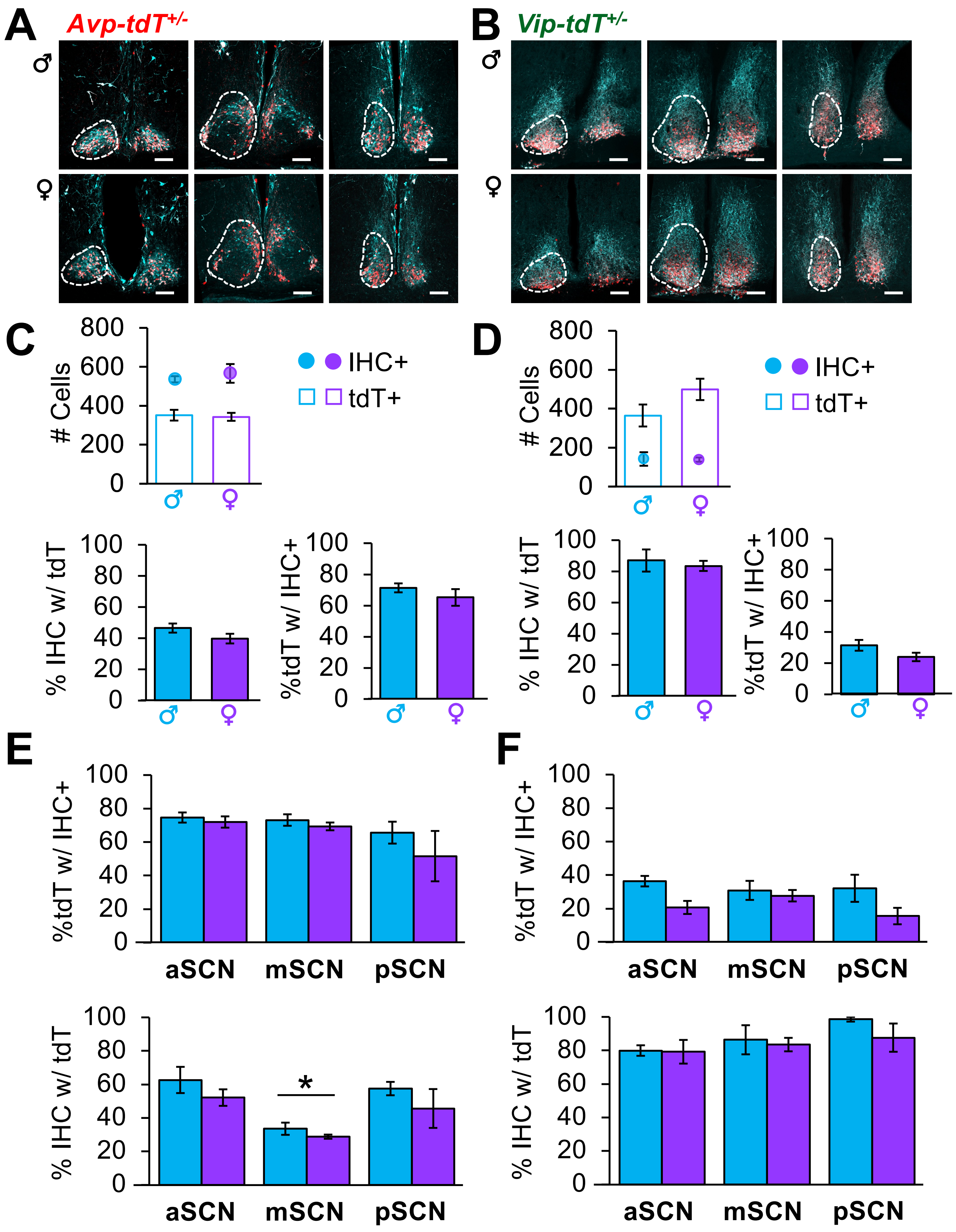

A
Avp-tdT+/-
B
Vip-tdT+/-
♂
♀
C
D
Cells
IHC+
tdT+
% IHC w/ tdT
%tdT w/ IHC+
E
F
aSCN
mSCN
pSCN
*

Figure S5

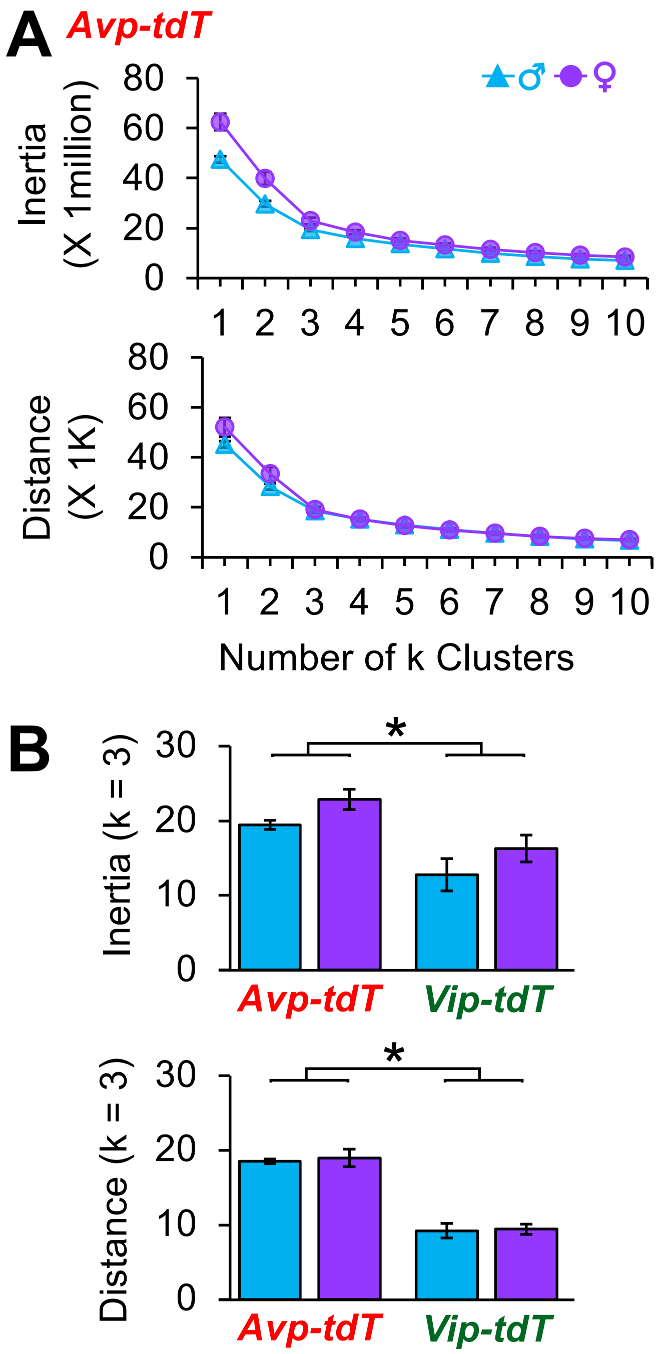
A
Avp-tdT
Vip-tdT
♂
♀
Inertia (X 1million)
Distance (X 1K)
Number of k Clusters
B
Inertia (k = 3)
Distance (k = 3)
*
Avp-tdT
Vip-tdT

Figure S6

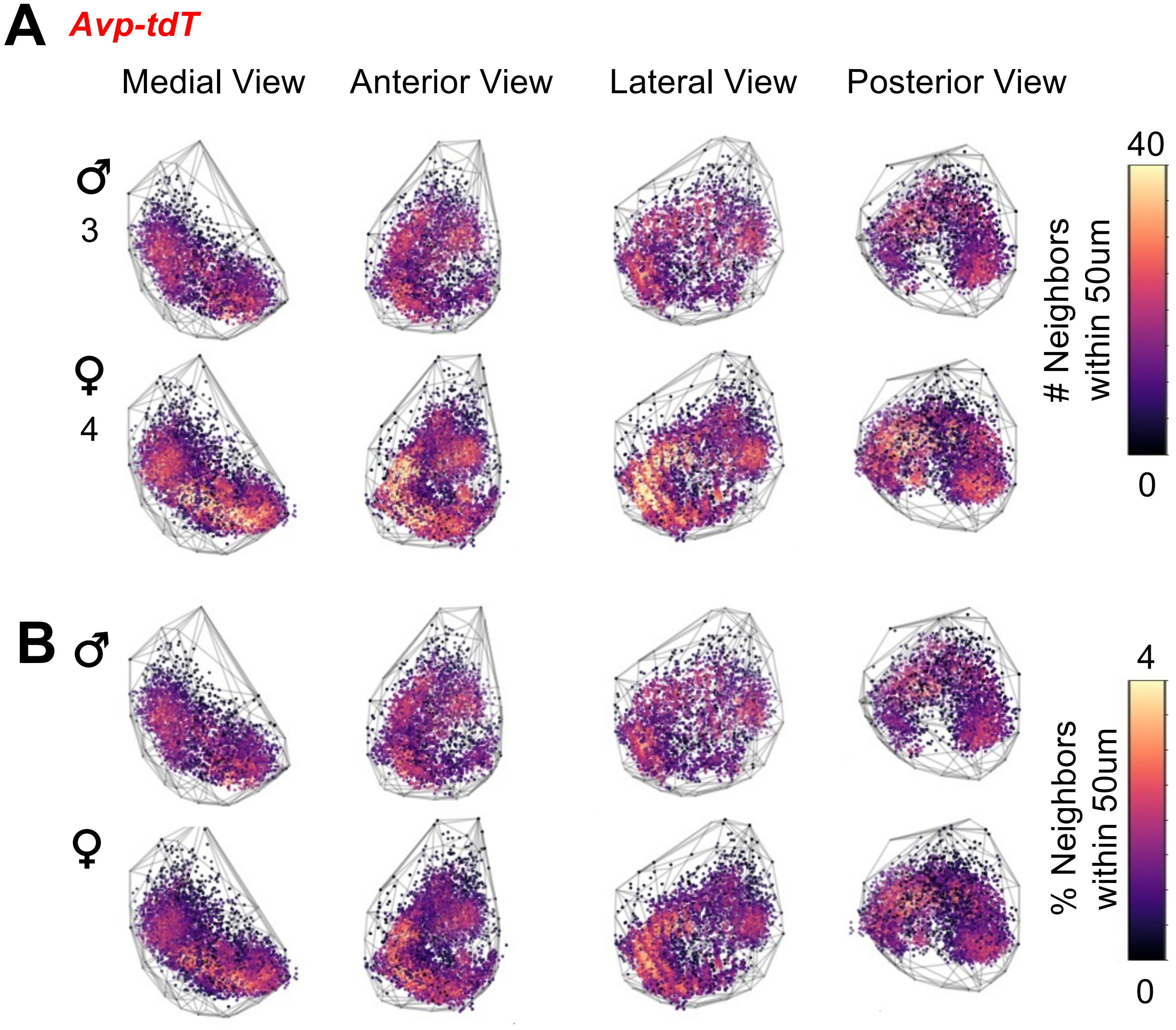
A
Avp-tdT
Medial View
Anterior View
Lateral View
Posterior View
♂
3
♀
4
40
Neighbors within 50um
0
B
♂
♀
4
% Neighbors within 50um
0

Figure S7

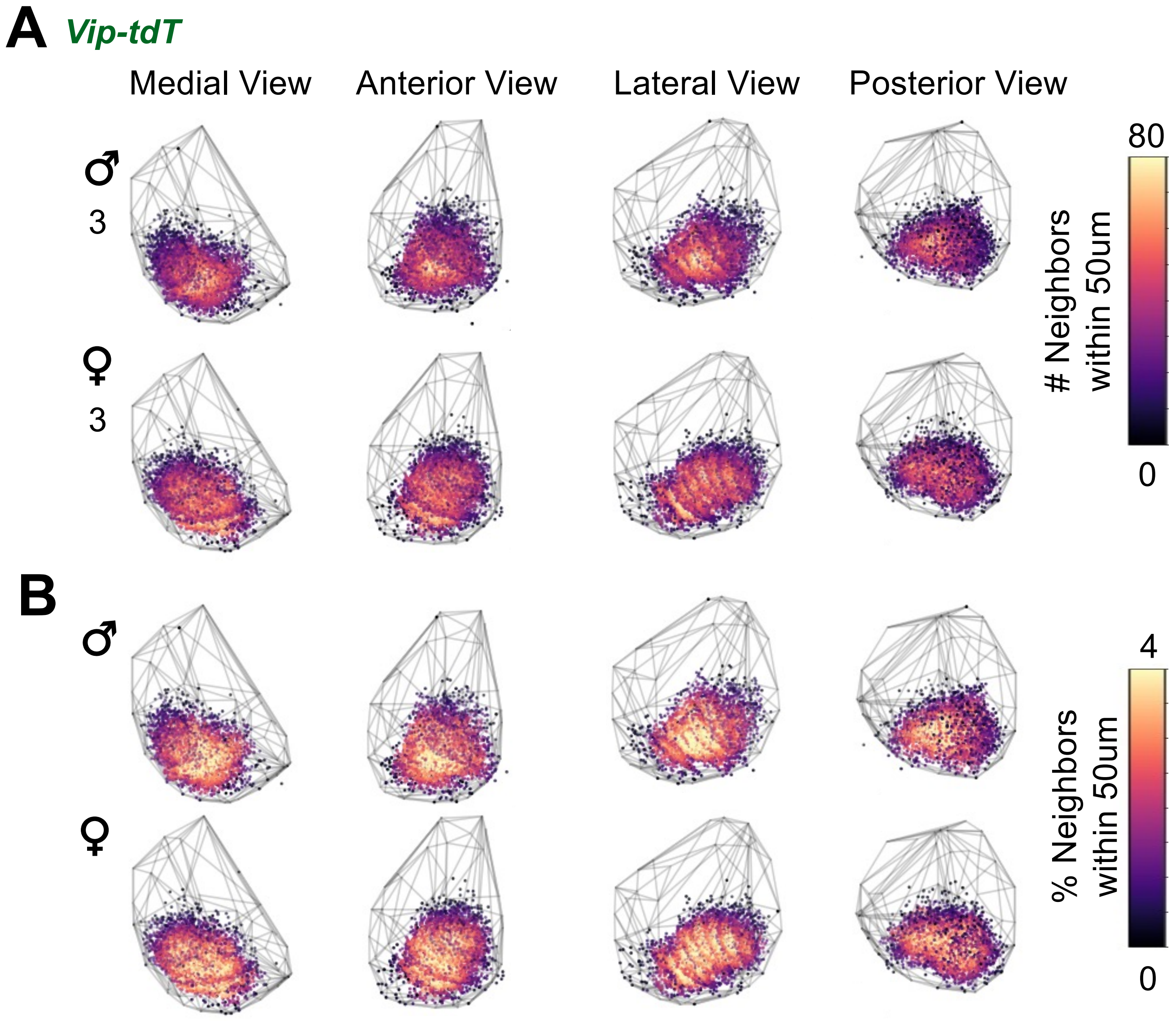
A
Vip-tdT
Medial View
Anterior View
Lateral View
Posterior View
♂
3
♀
3
80
Neighbors within 50um
0
B
♂
♀
4
% Neighbors within 50um
0

Figure S8

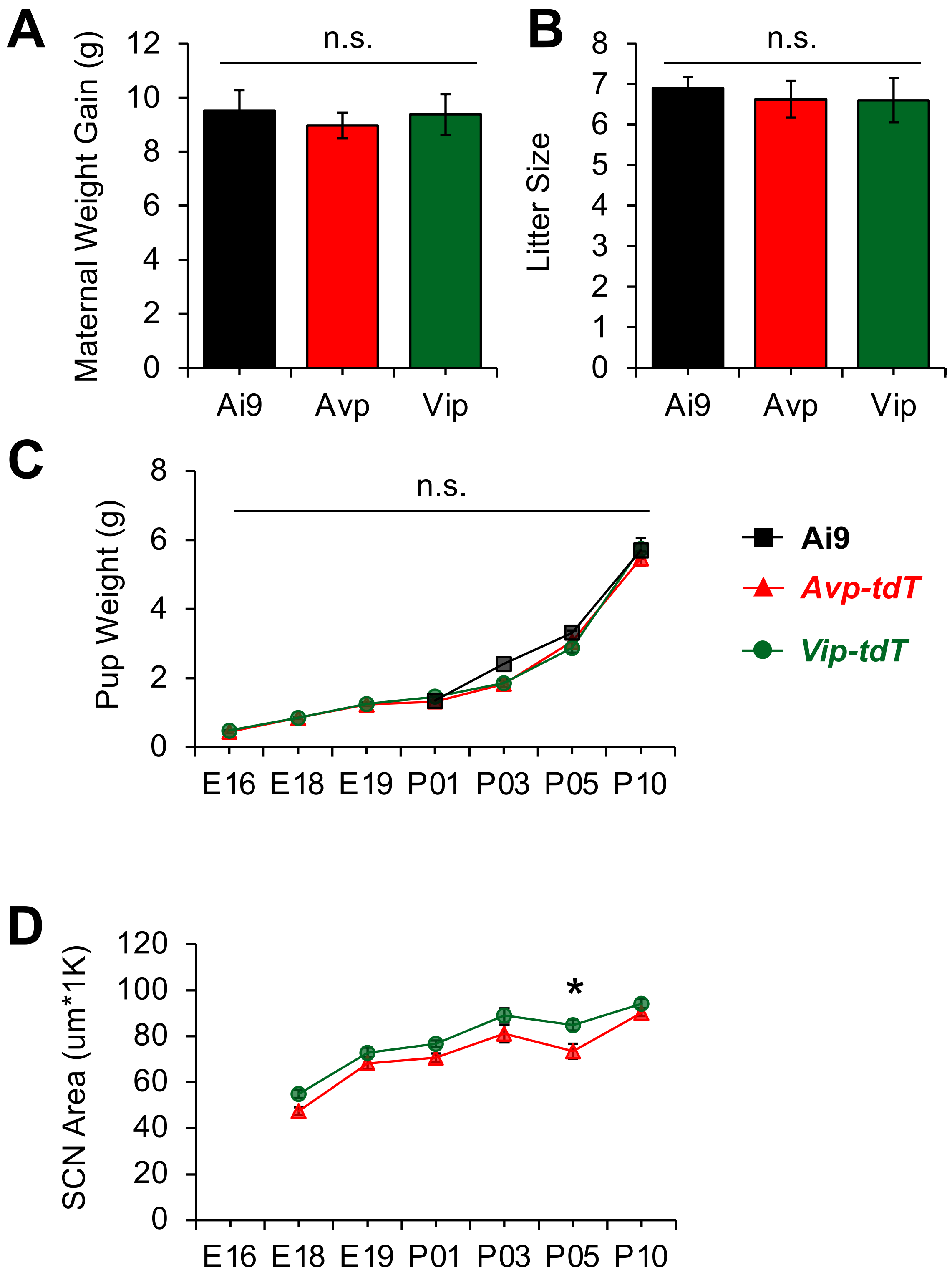

A
Maternal Weight Gain (g)
n.s.
0
2
4
6
8
10
12
Ai9
Avp
Vip
B
Litter Size
n.s.
0
1
2
3
4
5
6
7
8
Ai9
Avp
Vip
C
Pup Weight (g)
n.s.
0
2
4
6
8
E16
E18
E19
P01
P03
P05
P10
Ai9
Avp-tdT
Vip-tdT
D
SCN Area (um*1K)
*
0
20
40
60
80
100
120
E16
E18
E19
P01
P03
P05
P10

Figure S9

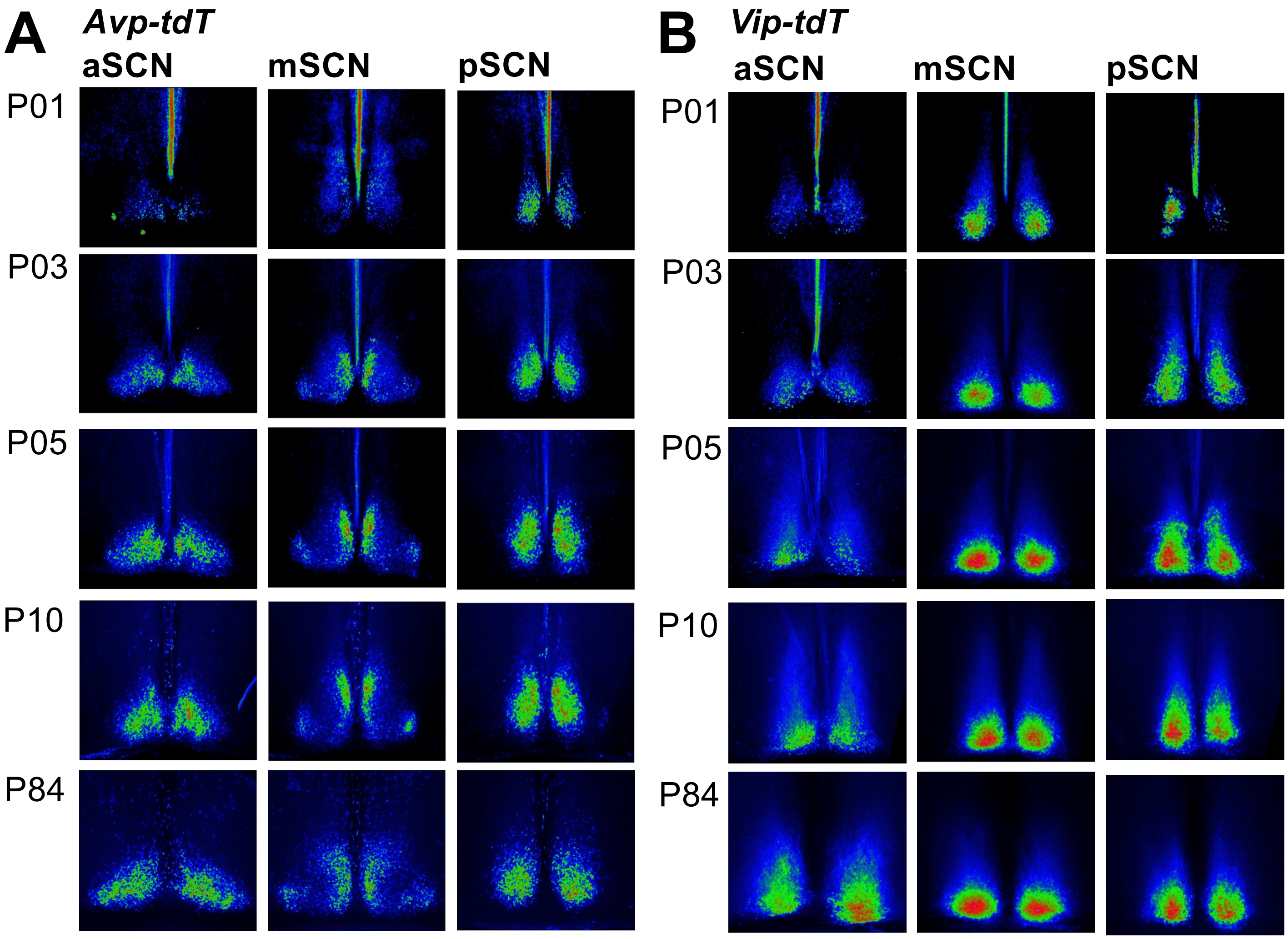
A
Avp-tdT
aSCN
mSCN
pSCN
P01
P03
P05
P10
P84
B
Vip-tdT
aSCN
mSCN
pSCN
P01
P03
P05
P10
P84

Figure S10

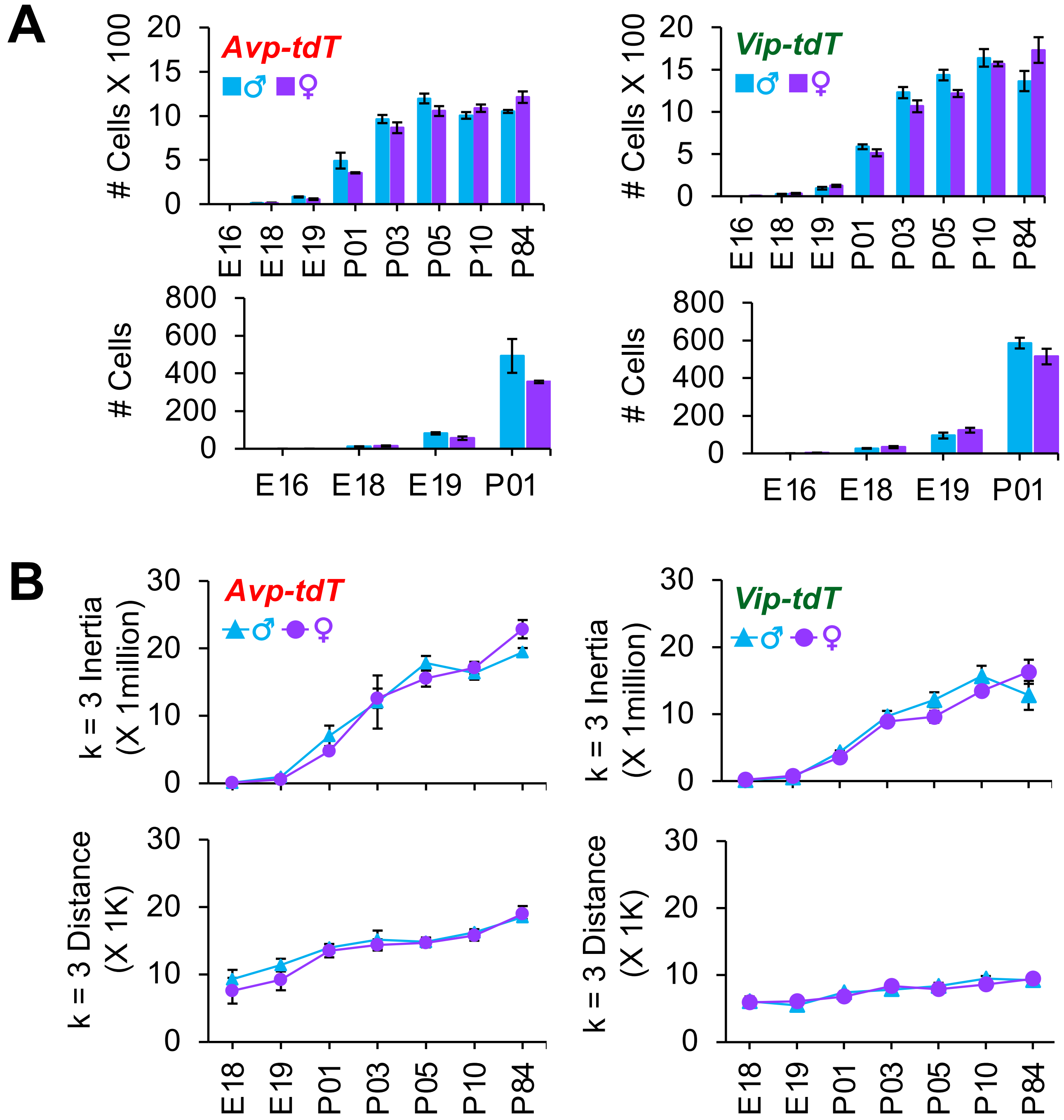
A
Avp-tdT
Vip-tdT
♂ ♀
Cells X 100
Cells
E16 E18 E19 P01 P03 P05 P10 P84
E16 E18 E19 P01
B
k = 3 Inertia (X 1million)
k = 3 Distance (X 1K)
E18 E19 P01 P03 P05 P10 P84

Figure S11
