# Supplementary figures and images for: Developmental patterning of peptide transcription in the central circadian clock in both sexes
Source: Front Neurosci. 2023 May 19;17:1177458. doi: 10.3389/fnins.2023.1177458 (PMC10235759; doi:10.3389/fnins.2023.1177458)

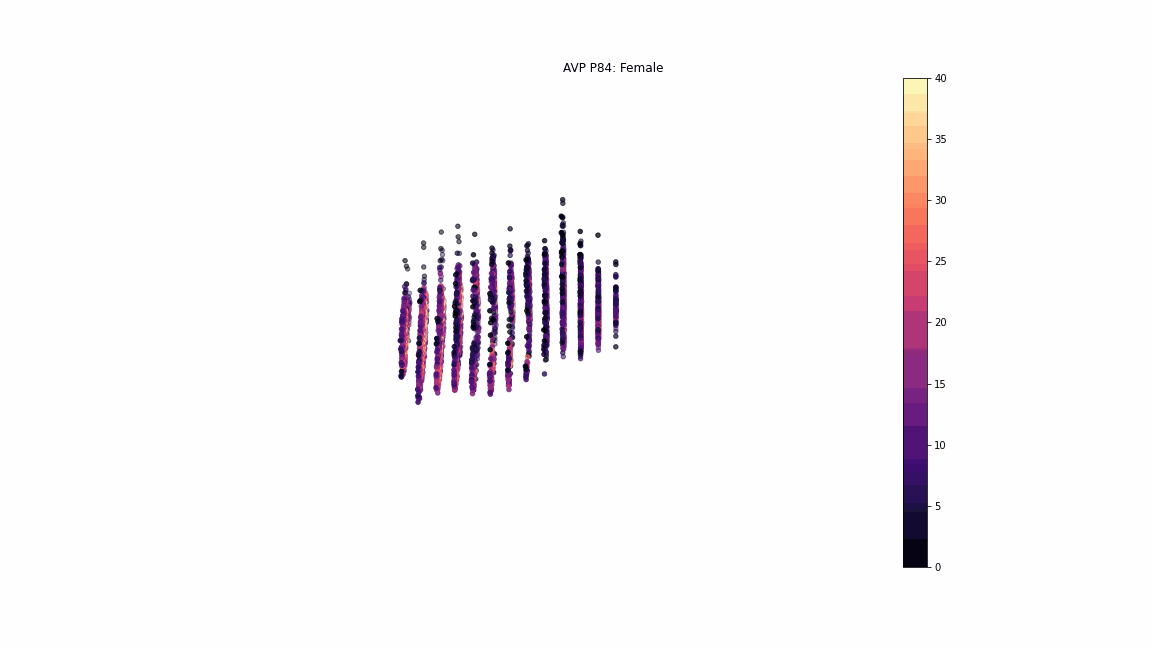

Supplement: Supplementary file 2 [file Data_Sheet_2.ZIP › SupplementalVideos/Video S3.gif]

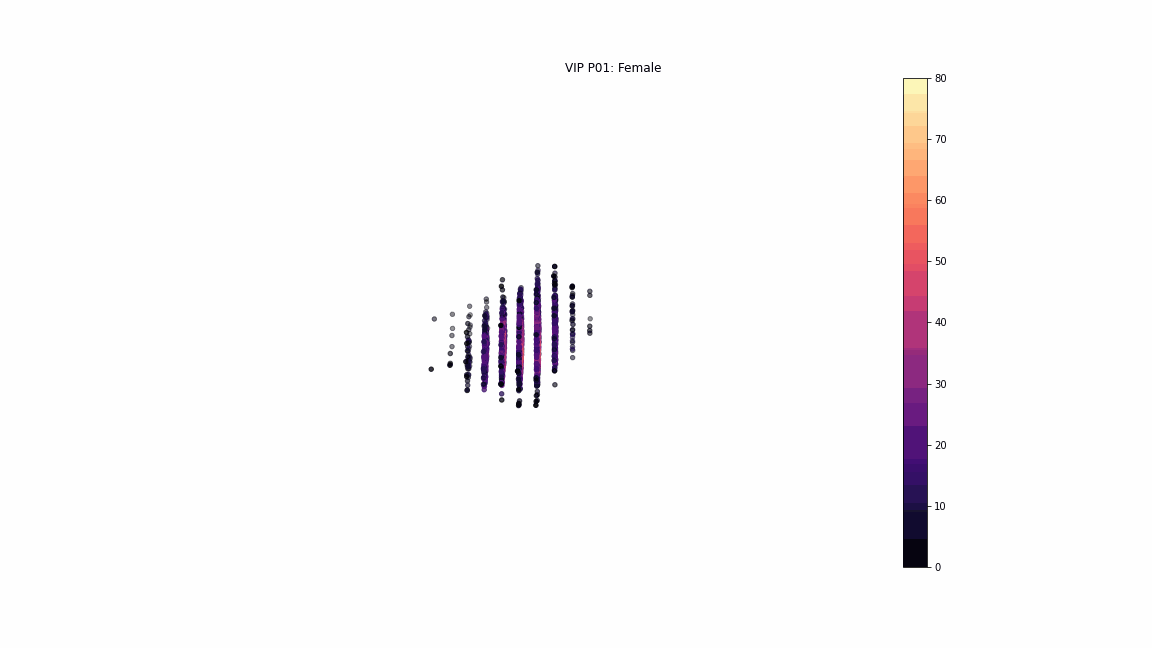

Supplement: Supplementary file 2 [file Data_Sheet_2.ZIP › SupplementalVideos/Video S11.gif]

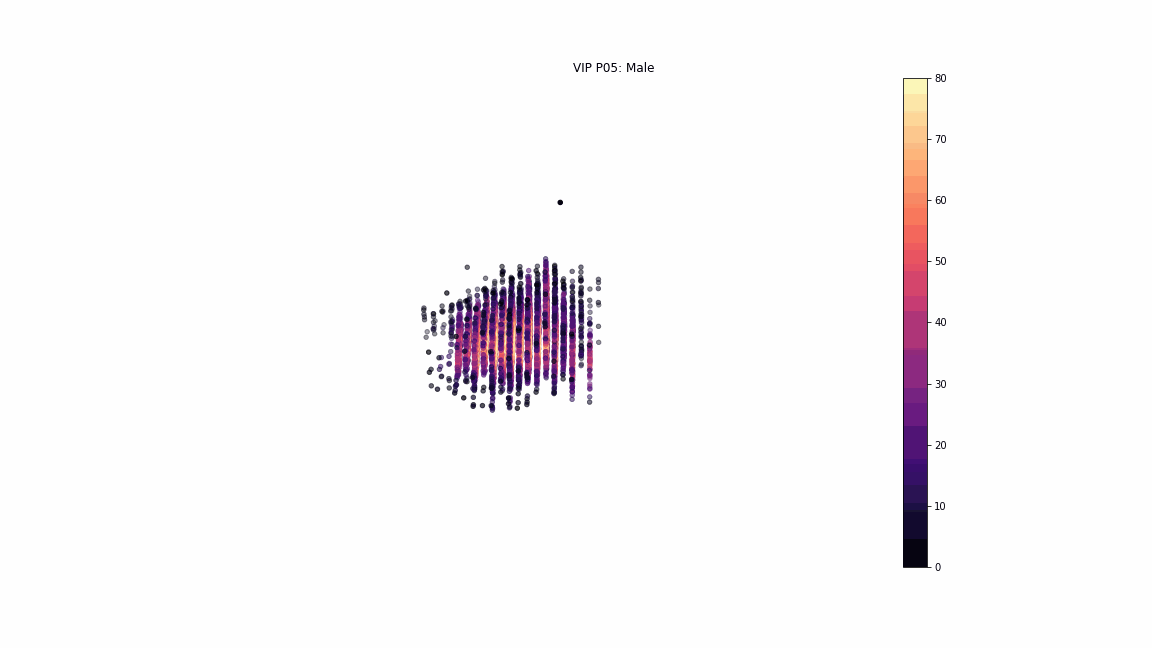

Supplement: Supplementary file 2 [file Data_Sheet_2.ZIP › SupplementalVideos/Video S10.gif]

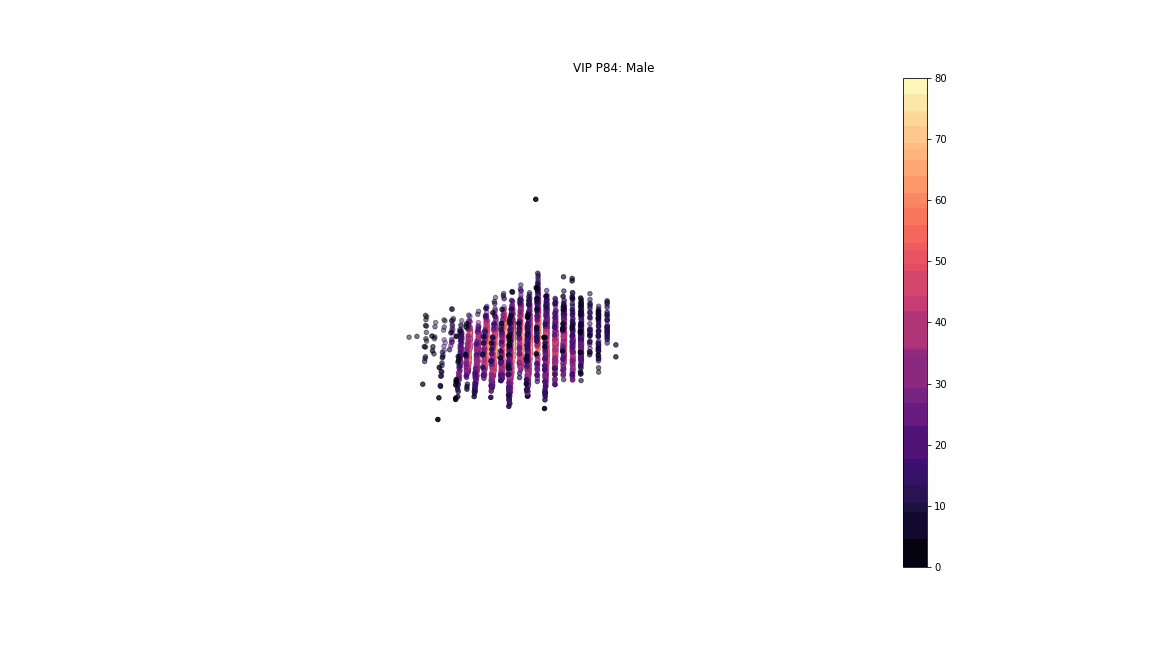

Supplement: Supplementary file 2 [file Data_Sheet_2.ZIP › SupplementalVideos/Video S2.gif]

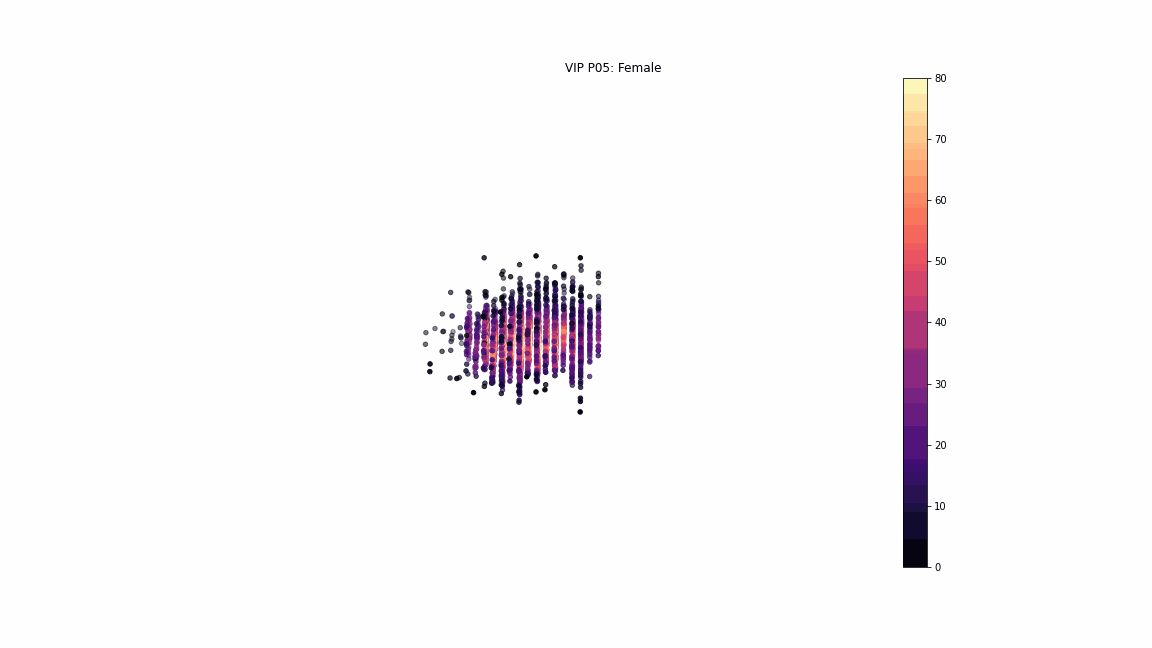

Supplement: Supplementary file 2 [file Data_Sheet_2.ZIP › SupplementalVideos/Video S12.gif]

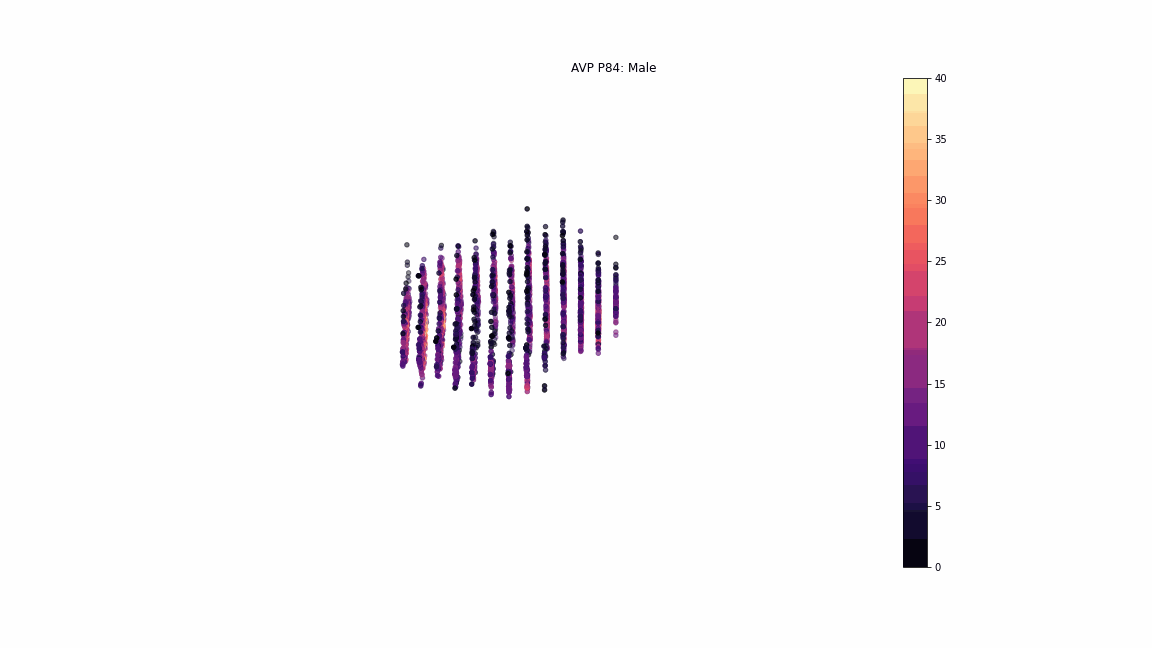

Supplement: Supplementary file 2 [file Data_Sheet_2.ZIP › SupplementalVideos/Video S1.gif]

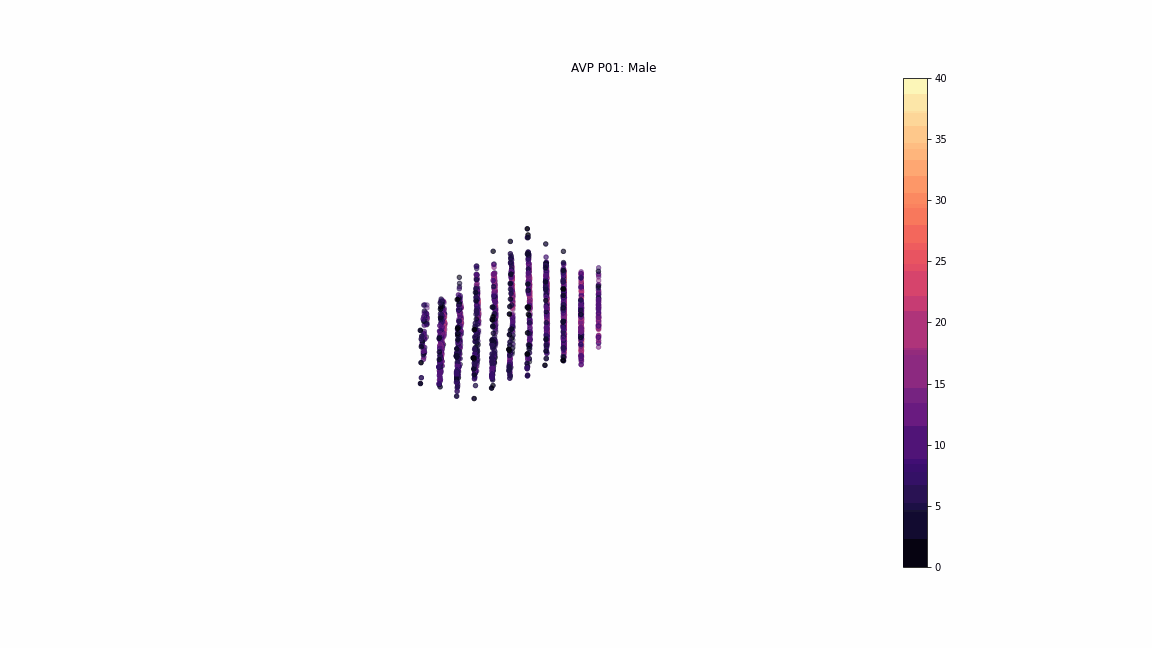

Supplement: Supplementary file 2 [file Data_Sheet_2.ZIP › SupplementalVideos/Video S5.gif]

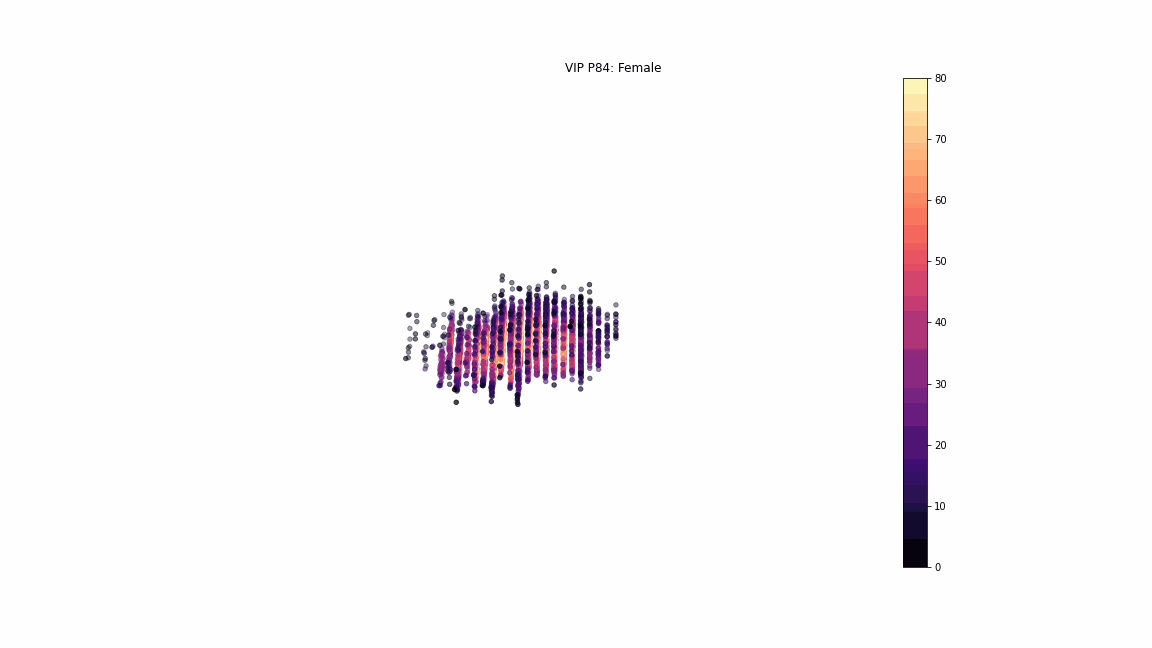

Supplement: Supplementary file 2 [file Data_Sheet_2.ZIP › SupplementalVideos/Video S4.gif]

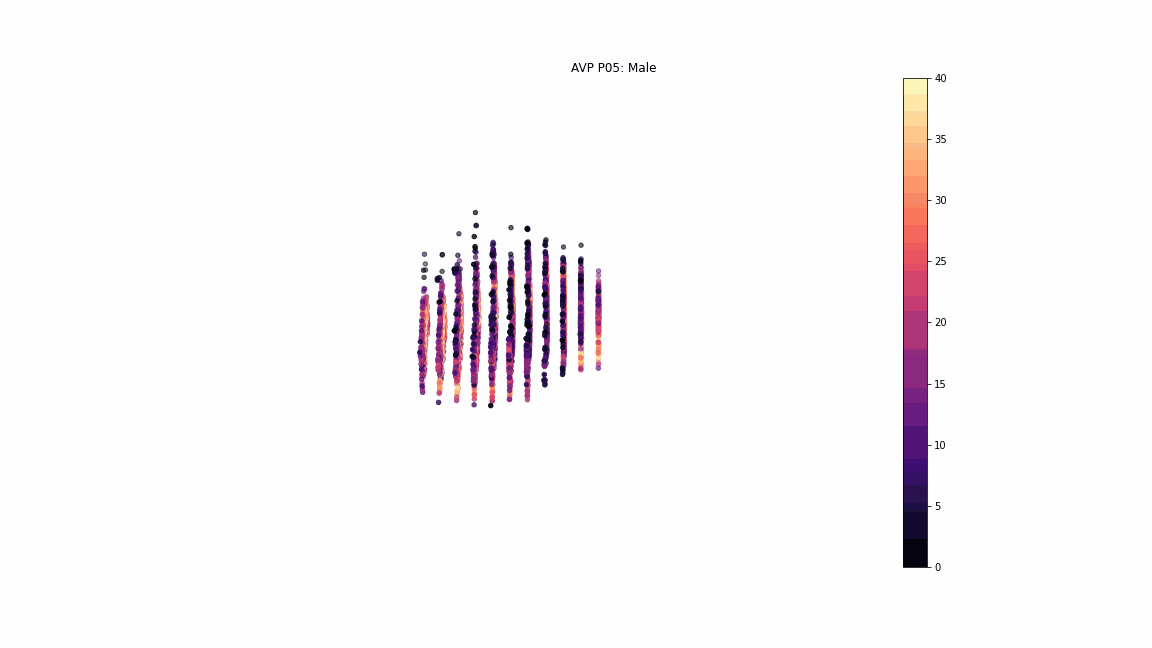

Supplement: Supplementary file 2 [file Data_Sheet_2.ZIP › SupplementalVideos/Video S6.gif]

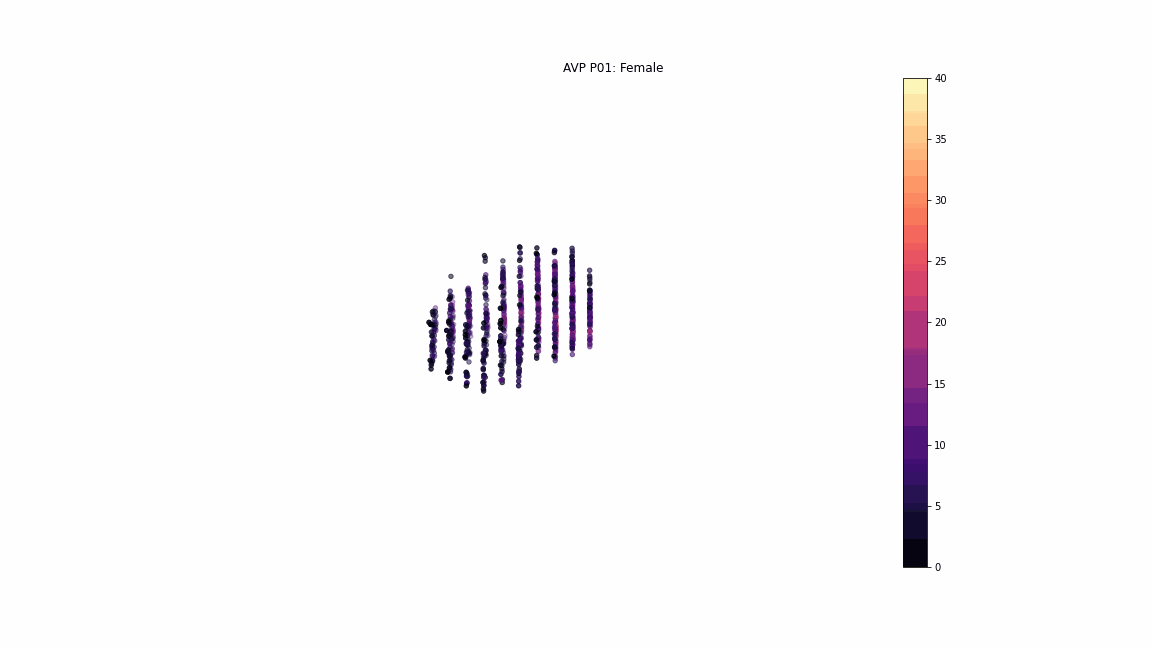

Supplement: Supplementary file 2 [file Data_Sheet_2.ZIP › SupplementalVideos/Video S7.gif]

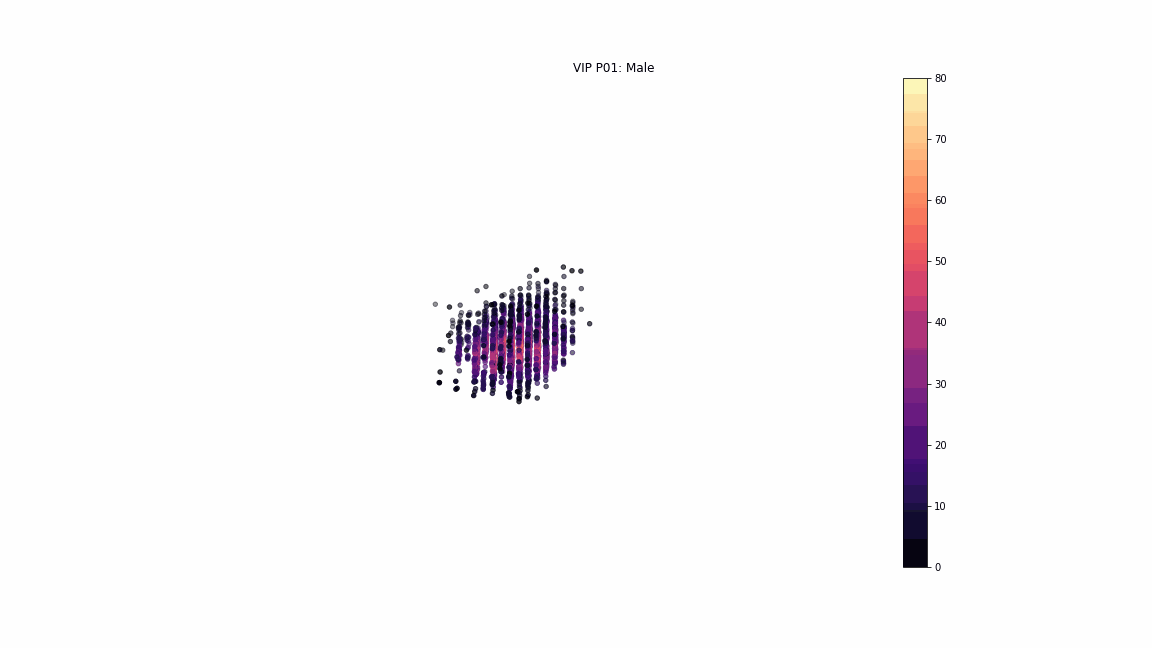

Supplement: Supplementary file 2 [file Data_Sheet_2.ZIP › SupplementalVideos/Video S9.gif]

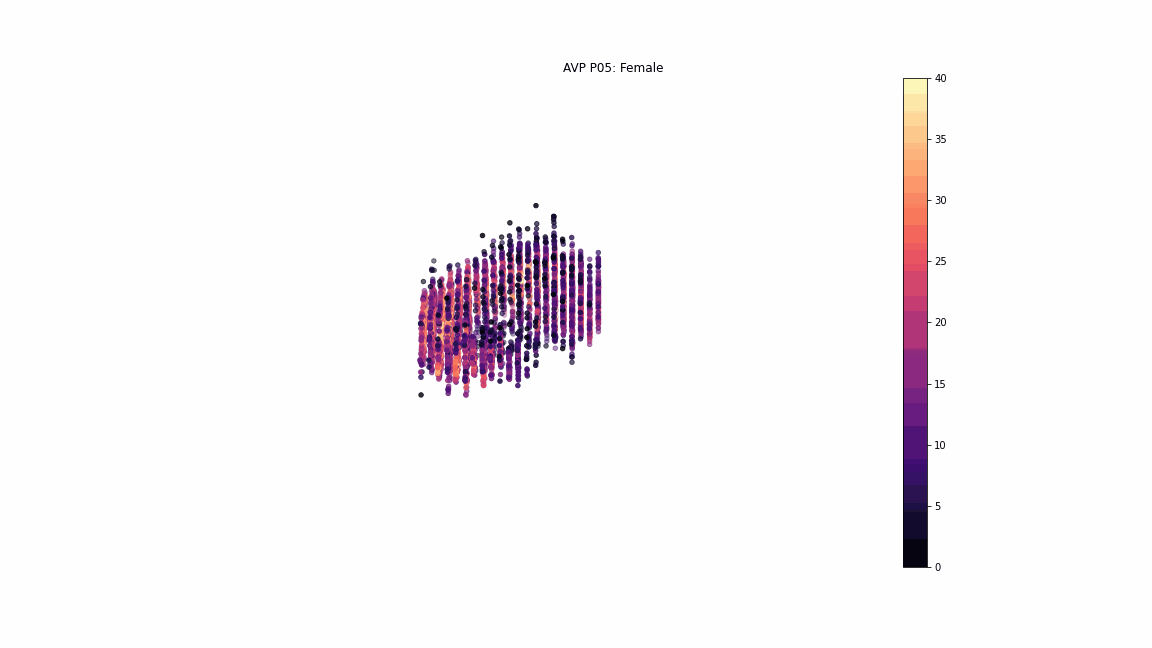

Supplement: Supplementary file 2 [file Data_Sheet_2.ZIP › SupplementalVideos/Video S8.gif]
